# Distinct conformational changes occur within the intrinsically unstructured pro‐domain of pro‐Nerve Growth Factor in the presence of ATP and Mg^2+^

**DOI:** 10.1002/pro.4563

**Published:** 2023-02-01

**Authors:** Francesca Paoletti, Sonia Covaceuszach, Alberto Cassetta, Antonio N. Calabrese, Urban Novak, Petr Konarev, Jože Grdadolnik, Doriano Lamba, Simona Golič Grdadolnik

**Affiliations:** ^1^ Laboratory for Molecular Structural Dynamics, Theory Department National Institute of Chemistry Ljubljana Slovenia; ^2^ Institute of Crystallography—C.N.R.—Trieste Outstation Trieste Italy; ^3^ School of Molecular and Cellular Biology, Astbury Centre for Structural Molecular Biology University of Leeds Leeds UK; ^4^ A.V. Shubnikov Institute of Crystallography of Federal Scientific Research Centre “Crystallography and Photonics” Russian Academy of Sciences Moscow Russia; ^5^ Interuniversity Consortium “Biostructures and Biosystems National Institute” Rome Italy

**Keywords:** conformational rearrangement of the Intrinsically Unstructured Domain (IUD), Hydrogen‐Deuterium eXchange‐Mass Spectrometry (HDX‐MS), intermolecular interactions, NMR spectroscopy, proNGF, Small Angle X‐ray Scattering (SAXS)

## Abstract

Nerve growth factor (NGF), the prototypical neurotrophic factor, is involved in the maintenance and growth of specific neuronal populations, whereas its precursor, proNGF, is involved in neuronal apoptosis. Binding of NGF or proNGF to TrkA, p75^NTR^, and VP10p receptors triggers complex intracellular signaling pathways that can be modulated by endogenous small‐molecule ligands. Here, we show by isothermal titration calorimetry and NMR that ATP binds to the intrinsically disordered pro‐peptide of proNGF with a micromolar dissociation constant. We demonstrate that Mg^2+^, known to play a physiological role in neurons, modulates the ATP/proNGF interaction. An integrative structural biophysics analysis by small angle X‐ray scattering and hydrogen‐deuterium exchange mass spectrometry unveils that ATP binding induces a conformational rearrangement of the flexible pro‐peptide domain of proNGF. This suggests that ATP may act as an allosteric modulator of the overall proNGF conformation, whose likely distinct biological activity may ultimately affect its physiological homeostasis.

## 1 INTRODUCTION

Neurotrophins (NTs) constitute a family of growth factors with multiple roles in the development, differentiation, maintenance, and repair of the central and peripheral nervous systems (CNS and PNS). Nerve growth factor (NGF) is a prototypical NT, produced as a precursor (proNGF) which is cleaved by furin in the endoplasmic reticulum (Shooter, 2001). Both proteins are active as homodimers. proNGF is involved in neuronal apoptosis (Lee et al., 2001) and an imbalance in proNGF/NGF levels is associated with learning and memory deficits (Allard et al., 2012; Buttigieg et al., 2007; Counts & Mufson, 2005). Measuring the relative levels of proNGF/NGF could therefore provide a new potential diagnostic biomarker for neurodegenerative disease (Malerba et al., 2016). Each NT can signal through two different types of cell surface receptors—the specific Trk tyrosine kinase receptors and the p75^NTR^ pan‐neurotrophin receptor (Hempstead, 2006). Proneurotrophins (proNTs) bind with different affinities to each receptor, as well as to receptors of the VP10p family (sortilin, SorCS2, SorCS3) (Hempstead, 2014).

The pro‐peptide region of proNGF is an intrinsically unstructured domain (IUD) (Covaceuszach et al., 2021; Paoletti et al., 2009). Small angle X‐ray scattering (SAXS) provided the first glimpse into the 3D structure of proNGF (Covaceuszach et al., 2021; Paoletti et al., 2009), and an integrative structural biophysics approach captured specific interactions encompassing the pro‐peptide and the mature domain (Paoletti et al., 2011; Trabjerg et al., 2017; Yan et al., 2019). The molecular determinants of proNGF interactions with its receptors have also been reported (Feng et al., 2010; Trabjerg et al., 2017). Naturally occurring small molecules in vivo have been proposed as physiological endogenous modulators of NGF signaling. Namely, the energy‐carrying molecule ATP has been proposed to play a key role in TrkA receptor signaling mediated by NGF (Hasche et al., 2010; Paoletti & Lamba, 2021; Paoletti et al., 2021). However, the underlying molecular binding mechanism of ATP to proNGF has remained unknown.

Here, using differential scanning fluorimetry (DSF), isothermal titration calorimetry (ITC), ^1^H saturation transfer difference NMR (STD‐NMR), and transferred NOESY NMR (trNOESY) measurements, we have found that ATP binds to recombinant human proNGF (rh‐proNGF). Furthermore, a combination of SAXS, hydrogen‐deuterium exchange mass spectrometry (HDX‐MS), and limited proteolysis, provides evidence that ATP binding induces conformational rearrangements of rh‐proNGF, likely confined to the IUD pro‐peptide. We also demonstrate that Mg^2+^ fine‐tunes the binding affinity of ATP to rh‐proNGF. Finally, we investigated by surface plasmon resonance (SPR) experiments the effects of the ATP on the binding affinity of rh‐proNGF versus its receptors. Our data suggest that ATP acts as an allosteric modulator of the overall proNGF conformation with likely functional effects on its physiological function.

## 2 RESULTS

**FIGURE 1:**
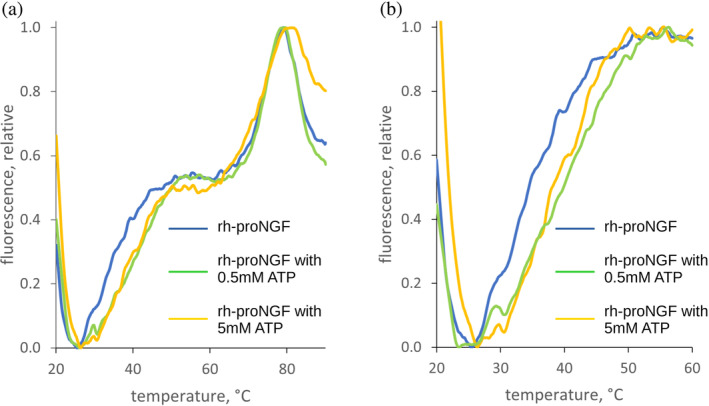
DSF melting profiles of rh‐proNGF in the absence and presence of two different concentrations of ATP (a) normalized according to the unfolding transition of mature rhNGF; (b) normalized according to the unfolding transition of the pro‐peptide domain. DSF, differential scanning fluorimetry; NGF, nerve growth factor

First, to determine if ATP binds to rh‐proNGF, we conducted DSF measurements in the absence and presence of ATP (Figure 1). Both DSF profiles show two transitions, consistent with previous measurements of unbound rh‐proNGF (Covaceuszach et al., 2021; Paoletti et al., 2011). The lower temperature transition corresponds to the unfolding of the flexible pro‐peptide domain, whereas the higher temperature transition corresponds to unfolding of the mature domain, as confirmed by DSF experiments on mature rh‐NGF (Paoletti et al., 2015). In the presence of ATP, the unfolding temperature for the mature domain of rh‐proNGF does not change, while the unfolding temperature for the pro‐peptide domain is increased by 4.75 ± 0.15°C, suggesting that ATP may bind to and/or stabilize the pro‐peptide domain of rh‐proNGF (Figure 1).

We next confirmed that ATP could bind to rh‐proNGF by performing ^1^H STD‐NMR experiments (Figure S1) that monitored the difference in intensity of the ATP proton signals due to saturation transfer in the presence of rh‐proNGF. All ATP proton signals show a change in intensity due to saturation transfer, thus confirming binding to rh‐proNGF. The H2 proton of the adenine moiety shows a significantly higher STD amplification factor, indicating that the adenine group is in stronger contact with rh‐proNGF than the sugar moiety of ATP (Figure S1). These observations were validated by using trNOESY. Negative NOEs with the same sign as the diagonal peaks were observed among the ATP protons in the trNOESY spectrum, indicating that ATP adapts to the protein surface. NOEs were identified between the H8 proton in the adenine moiety and H1’, H2’, and H3’ protons in the sugar moiety (Figure S2). The intensities of H8‐H1’, H8‐H2’ and H8‐H3’ NOEs are comparable, opposite to what expected from the distances between these protons within ATP structure. This behavior points to ATP binding in more than one conformational pose with distinct relative orientations of the adenine and sugar moieties. This agrees with the reported conformational flexibility of ATP upon protein binding (Nishizawa et al., 2021).

**FIGURE 2:**
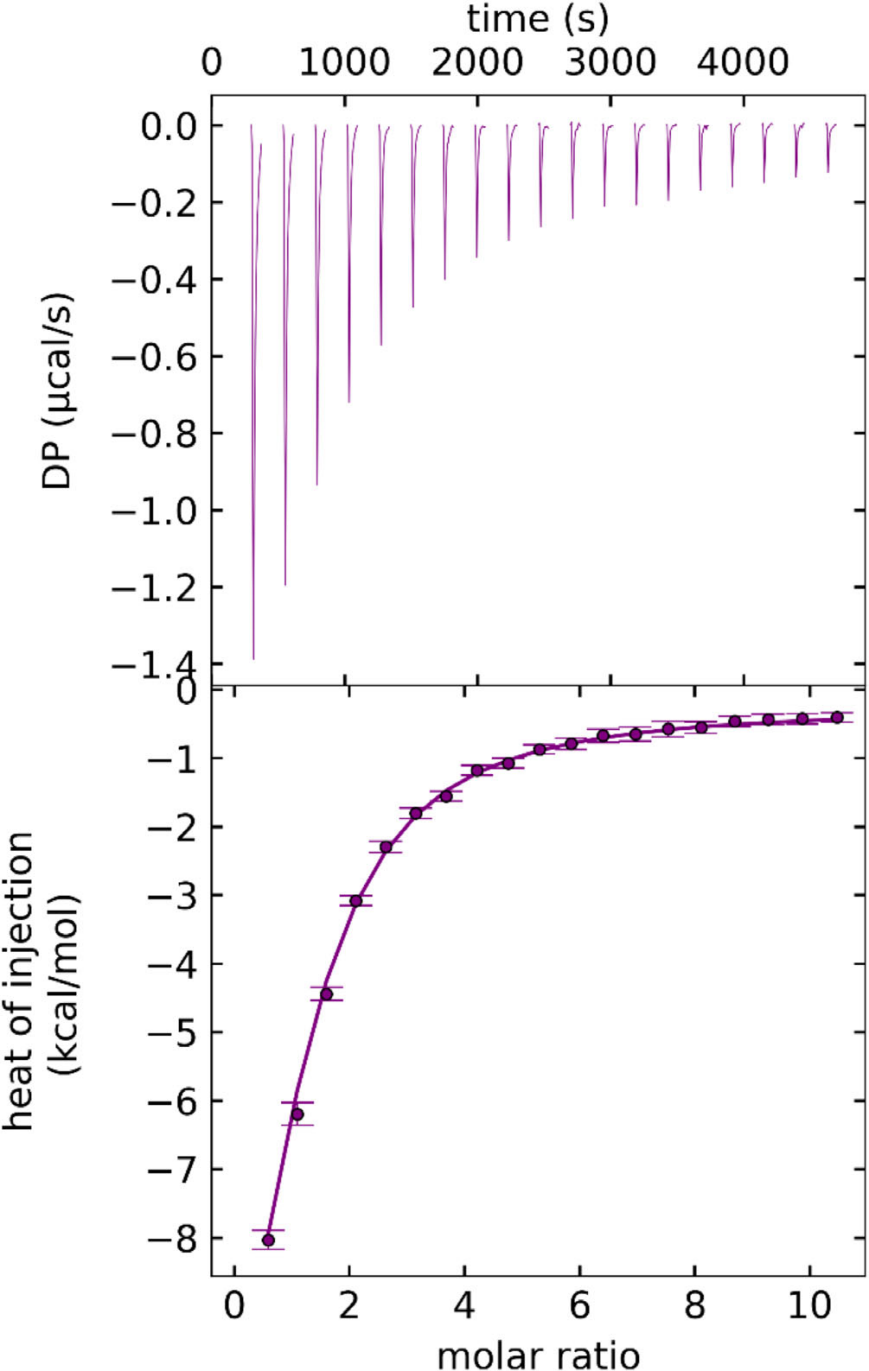
Interaction of rh‐proNGF with ATP by ITC. Thermograms and binding isotherms (upper and lower panel, respectively) of a representative ATP/rh‐proNGF titration (2 mM ATP, 20 μM rh‐proNGF with respect to the dimer). Thermodynamic parameters of rh‐proNGF/ATP binding as determined by ITC isotherm fitting are reported in Table 1. ITC, isothermal titration calorimetry; NGF, nerve growth factor

The affinity of the ATP/rh‐proNGF interaction was then determined using ITC (Figure 2 and Table 1). The measured *K*_D_ of 39.7 μM is significantly lower than the previously reported *K*_D_ of 1.38 mM for the ATP/rhNGF interaction (Paoletti et al., 2021), underlining that the IUD pro‐peptide domain plays a role in increasing the binding affinity of ATP to rh‐proNGF.

**TABLE 1:** Best‐fit value obtained by global fitting of two independent ITC titrations of 2 mM ATP titrations into 20 μM rh‐proNGF (dimer)

ATP— > rh‐proNGF	Best‐fit value	68.3% confidence interval
*K*_D_ (μM)	39.7	35.7; 45.4
*ΔH* kcal mol^−1^	−19.0	−20.1; −17.9
*ΔS* cal mol^−1^ K^−1^	−40.9	

*Note*: Binding stoichiometry: ATP:rh‐proNGF = 1:1, with respect to rh‐proNGF protomer.

Abbreviations: ITC, isothermal titration calorimetry; NGF, nerve growth factor.

**FIGURE 3:**
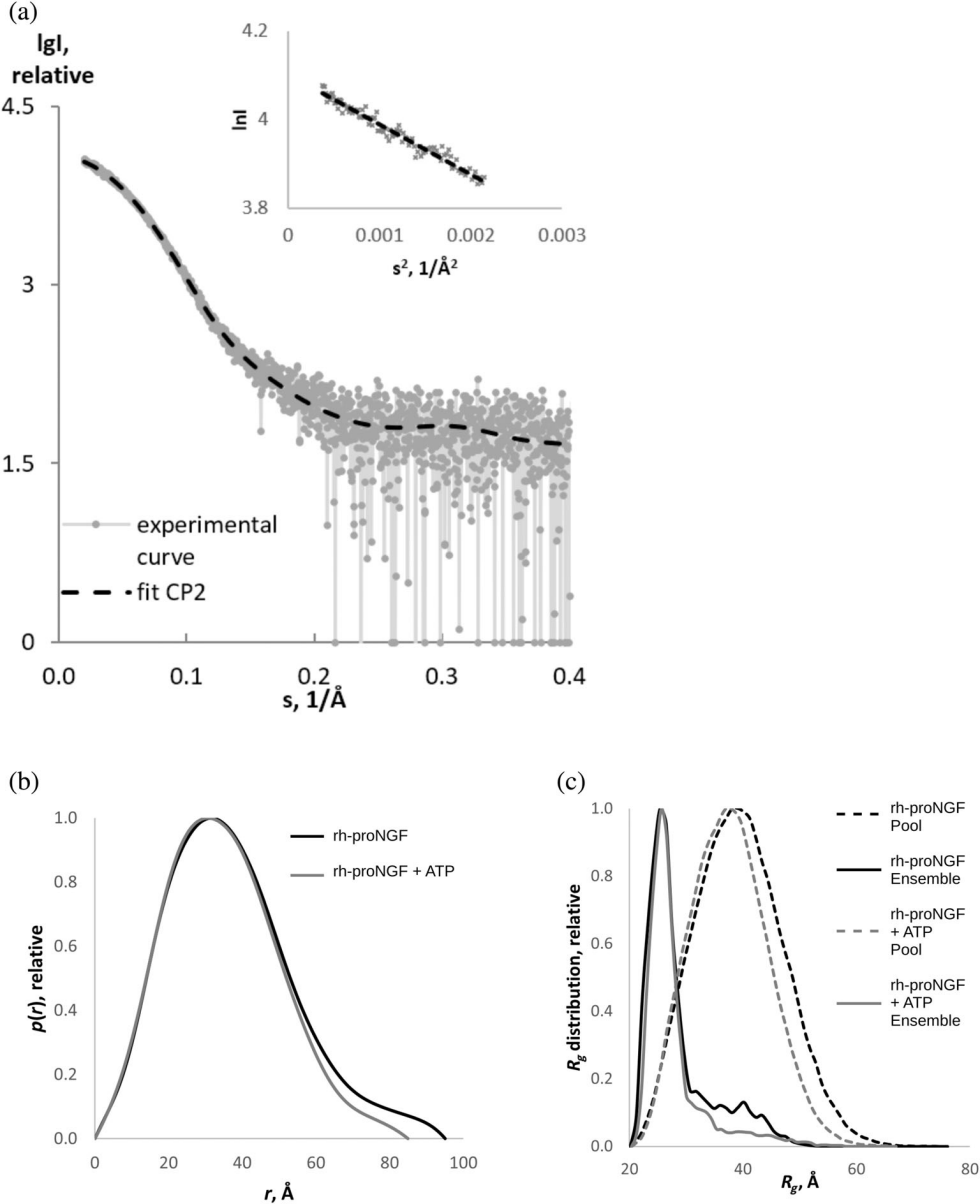
SAXS experimental profiles and data analysis of rh‐proNGF in the absence and presence of ATP. (a) Experimental SAXS data, measured for rh‐proNGF in presence of ATP, compared with the scattering calculated from the obtained high‐resolution 3D model of rhNGF (Covaceuszach et al., 2015) with added missing regions obtained by CORAL (Petoukhov et al., 2012) imposing a P2 symmetry (fit CP2). The plots display the logarithm of the scattering intensity as a function of momentum transfer (inset: corresponding Guinier plots). (b) Comparison of the distance distribution functions of rh‐proNGF in absence and presence of ATP. (c) *R*_g_ distributions obtained by EOM applying P2 symmetry for rh‐proNGF in the absence and presence of ATP. Dotted and solid lines show the distributions for the initial random pools of models and for the selected ensembles, respectively. NGF, nerve growth factor; SAXS, small angle X‐ray scattering

**FIGURE 4:**
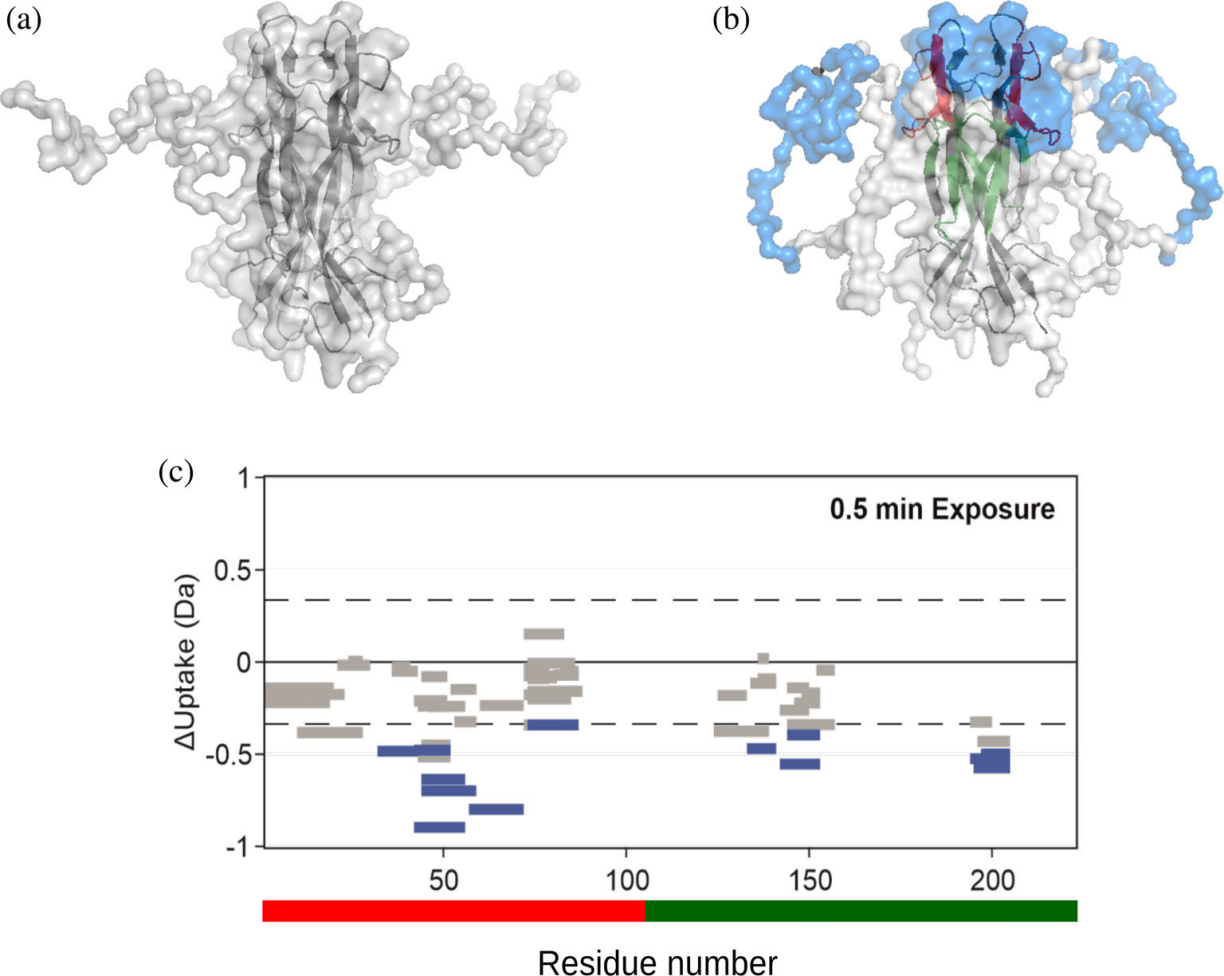
Conformational effects of ATP binding to rh‐proNGF by SAXS (a, b) and HDX‐MS (c). (a, b) Rigid body models of rh‐proNGF without (Covaceuszach et al., 2021) (a) or with ATP (b) obtained by CORAL (Petoukhov et al., 2012) (semitransparent surfaces) applying P2 symmetry. The high‐resolution 3D model of rhNGF (Covaceuszach et al., 2015) is shown as a gray cartoon. In (b) the semitransparent blue surfaces correspond to the peptides that were protected from deuterium exchange upon ATP binding. Residues identified as ATP binding sites on rhNGF by NMR(Paoletti et al., 2021) are colored in green (Site 1) and red (Site 2) on the ribbon representation of rhNGF in gray. Figures produced by Pymol. (c) Wood's plot shows the differences in deuterium uptake in rh‐proNGF after 0.5 min incubation in deuterium‐containing buffer, comparing rh‐proNGF alone with rh‐proNGF in the presence of ATP. The scheme of rh‐proNGF with the pro‐peptide in red and the mature NGF in green is shown in the bottom of the plot. Wood's plot was generated using Deuteros (Lau et al., 2021). Peptides colored in blue are protected from the exchange in the presence of ATP. Peptides with no significant difference between conditions, determined using a 99% confidence interval and a hybrid significance test (dotted line), are shown in gray. See Section 4 for experimental details. HDX‐MS, hydrogen‐deuterium exchange‐mass spectrometry; NGF, nerve growth factor; SAXS, small angle X‐ray scattering

Next, we validated structural and/or dynamical changes in rh‐proNGF upon ATP binding. Fourier transform infrared spectroscopy (FT‐IR) measurements ruled out any change in the overall secondary structure composition of rh‐proNGF upon ATP binding (Figure S3). To investigate the effect of ATP binding on the 3D shape of rh‐proNGF we exploited SAXS (Figure 3, Table S1, Table 2). The data indicates that, in the presence of ATP, the 3D reconstruction shows a more compact rh‐proNGF conformation if compared to that in the absence of ATP (Covaceuszach et al., 2021) (Table S1). The radius of gyration (*R*_g_) (Table 2) decreases in the presence of ATP from 28.8 ± 0.01 to 26.9 ± 0.05 Å and the profiles of the computed distance distribution functions *p*(*r*) (Figure 3c) point to a considerable decrease in the maximum dimensions (from 95 ± 3 to 85 ± 2 Å). Consistent with this, the resulting rigid body models, obtained using complexes with random loops (CORAL) (Petoukhov et al., 2012) (Figure 4a, b), show a more compact conformation of rh‐proNGF in the presence of ATP. Furthermore, ensemble optimization method (EOM) analysis (Bernadó et al., 2007) (Figure 3) clearly indicates the narrowing of the *R*_g_ within the distribution ensemble upon ATP binding. Indeed, in the presence of ATP the population characterized by an *R*_g_ in the range of 30–50 Å, drops from 25% to 18.5% of the total population, indicating a shift to a more compact and less flexible overall conformation. Combined, our data suggest that the IUD pro‐peptide rests on the surface of the mature rhNGF domain, and the IUD is likely stabilized by the bound ATP, as previously shown for other flexible proteins (Nishizawa et al., 2021).

**TABLE 2:** Summary of SAXS data: *Χ*_CP2_ and *Χ*_eom_ values for the fit from the crystallographic models with added N‐terminal pro‐domains obtained by CORAL and from EOM applying P2 symmetry, respectively

Sample	*R*_g_ (Å)	*D*_max_ (Å)	*V*_p_ (Å^3^)	*Χ*_CP2_	*Χ*_eom_
rh‐proNGF	28.8 ± 0.01	95 ± 3	80,300 ± 4000	1.472	1.007
rh‐proNGF + ATP	26.9 ± 0.05	85 ± 2	80,500 ± 4000	0.600	0.577

Abbreviations: NGF, nerve growth factor; SAXS, small angle X‐ray scattering.

**FIGURE 5:**
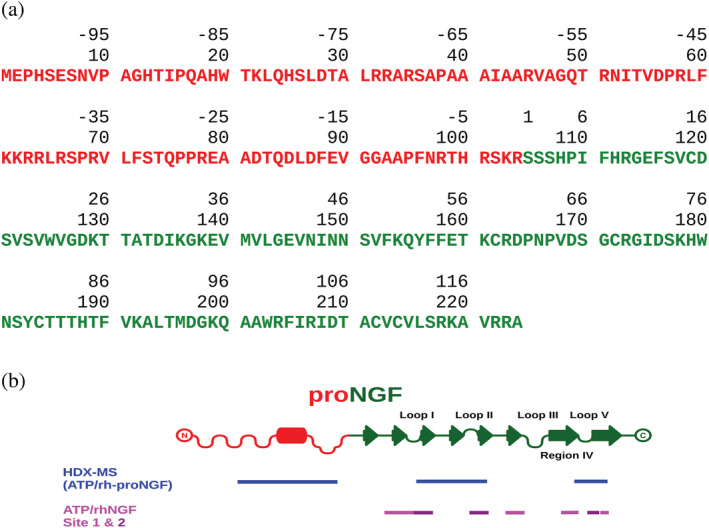
Schematic representation of rh‐proNGF and ATP binding. (a) Primary structure of rh‐proNGF. Red: pro‐peptide sequence; green: mature NGF. The double numbering scheme indicates the numbering whereby the start is on the first N‐terminal residue of mature hNGF (top); the numbering whereby the start is on the first residue of the rh‐proNGF (bottom). The number of positively charged residues (arginines, lysines, and histidines) is 23 out of 104 for the pro‐peptide and 21 out of 120 for rhNGF. (b) Schematic representation of the secondary structure elements of rh‐proNGF with the map of the peptides described in the text. Red: pro‐peptide; green: mature NGF. The red barrel on the pro‐peptide indicates the region with helical secondary structure propensity on rh‐proNGF according to MFDp2 prediction (Mizianty et al., 2013). The loops of mature NGF are labeled according to the literature (Ibáñez, 1995). Blue bars: peptides showing protection upon ATP binding according to HDX‐MS. Light and dark purple bars: binding peptides identified as Site 1 and Site 2 for the ATP/rhNGF binding, respectively (Paoletti et al., 2021). HDX‐MS, hydrogen‐deuterium exchange‐mass spectrometry; NGF, nerve growth factor

To further probe changes in the conformation and/or dynamics of rh‐proNGF upon ATP binding, we next performed differential HDX‐MS, by comparing the extent of deuterium uptake in rh‐proNGF in the absence and presence of ATP. The coverage of the protein sequence was good for the pro‐peptide but not optimal for the mature NGF (Figure S4) due to the cystine knot formed by the three disulfide bridges that are present in each of the mature NGF protomer, as previously reported (Trabjerg et al., 2017). Comparison of the extent of deuterium uptake in rh‐proNGF in the absence and presence of ATP revealed that peptides spanning residues of the pro‐peptide domain (residues 32–87—Figure 5a) were protected from exchange in the presence of ATP (Figure 4c). We cannot discriminate whether the protection is due to the direct contact of this region with ATP or with the mature domain of rhNGF. Nevertheless, we can confirm that this region undergoes a conformational/dynamical change upon ATP binding, resulting in decreased solvent exposure and/or increased hydrogen bonding. This finding agrees with previous reports observing a reduction in the extent of solvent exposure of proteins upon ATP binding (Nishizawa et al., 2021). Interestingly, the region that is protected from hydrogen exchange in the presence of ATP is highly conserved in NT pro‐peptides (Covaceuszach et al., 2021). It encompasses residues with a predicted helical propensity, being reported to have a functional role (Covaceuszach et al., 2021) (Figure 5). Additionally, residues 133–153 and 194–205 within the mature domain of rh‐proNGF (Figure 5) show protection from exchange in the presence of ATP. These residues overlap with the ATP binding site 2 on rhNGF as previously identified by 3D ^15^N‐NOESY‐HSQC NMR (Paoletti et al., 2021) (Figure 5b). Mapping these protected residues on the SAXS model of the ATP‐bound rh‐proNGF corroborates that they are confined to the same region of the molecule (Figure 4b). Furthermore, trypsin‐limited proteolysis experiments showed that rh‐proNGF is cleaved at a lower rate in the presence of an excess of ATP than in its absence (Figure S5). This result is in line with the SAXS and HDX‐MS experiments, whereby the increased compactness of the IUD pro‐domain of rh‐proNGF induced by ATP contributes to a lower accessibility of the cleavage sites on the IUD pro‐peptide.

To investigate the effects of ATP binding on the interaction of rh‐proNGF with sortilin, p75^NTR^, and TrkA receptors, we performed SPR experiments by immobilizing the receptors on the sensor chip and flowing rh‐proNGF with or without added ATP over the surface. The measurements were carried out in an experimental condition with a large excess of ATP over rh‐proNGF, aimed at shifting the binding equilibrium *versus* the formation of the ATP/rh‐proNGF complex. A quantitative analysis of the binding kinetics cannot be performed because of the strong influence of mass transport effects on the kinetics of binding to the surface, most likely due to the flexible nature of rh‐proNGF. Nevertheless, a qualitative analysis of the data was carried out by sensorgrams comparison based on curves' similarity and intensity assessments. The results show that rh‐proNGF can bind to all three of the receptors, but the influence of ATP on this binding differs in each case (Figure S6). Indeed, the ATP/rh‐proNGF adduct shows a weaker binding affinity than rh‐proNGF alone to sortilin. The opposite occurs for the p75^NTR^ and TrkA receptors. These different effects are likely the results of the conformational rearrangements, largely limited within the pro‐peptide domain, upon ATP binding to rh‐proNGF, which in turn affect the interacting surfaces with the receptors. This suggests that the role of ATP in modulating signaling is likely dependent on which of the receptor being involved.

**FIGURE 6:**
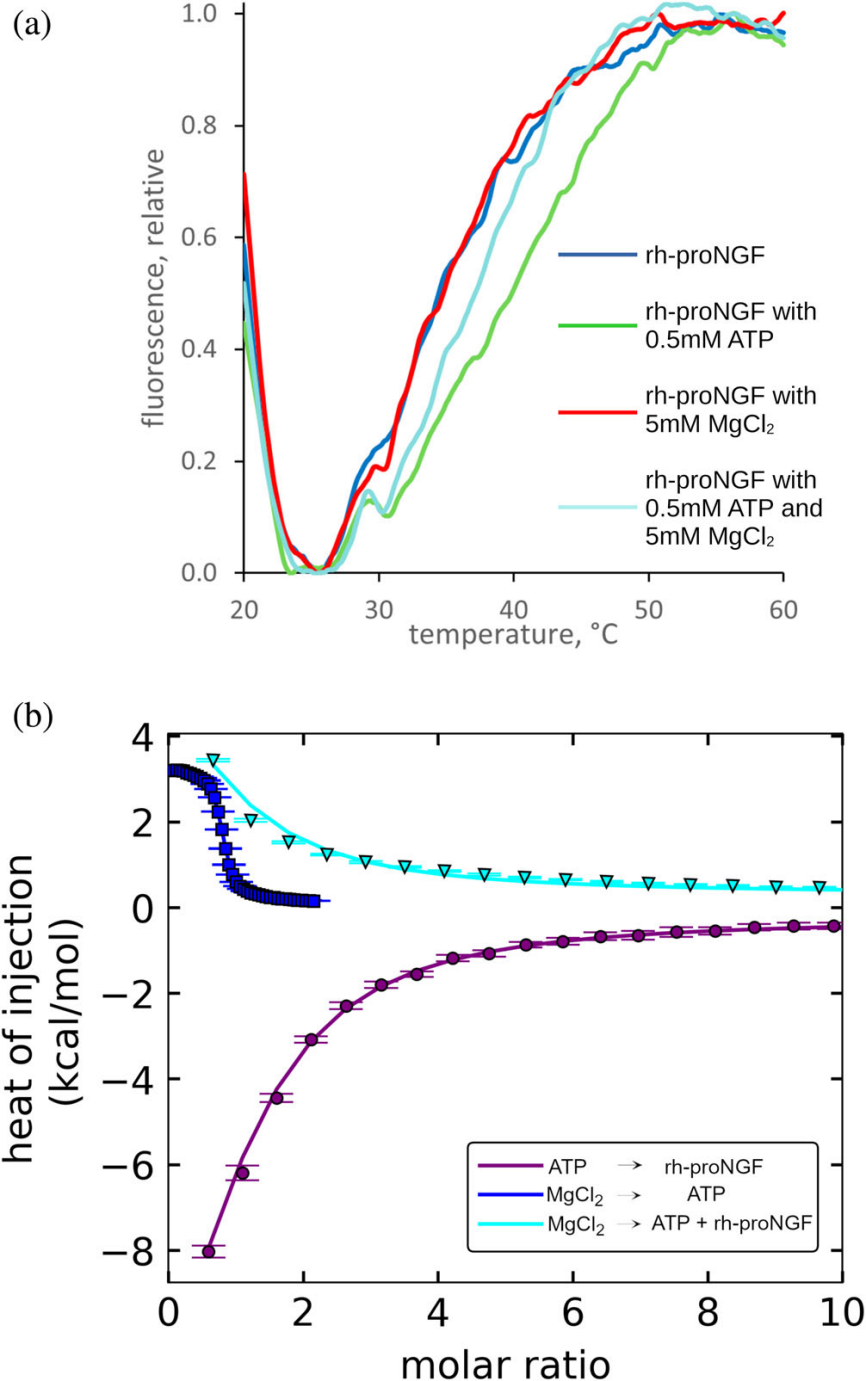
DSF and ITC demonstrate that Mg^2+^ modulates ATP interaction with rh‐proNGF. (a) Melting profiles of rh‐proNGF in presence of 0.5 mM ATP, in the absence and presence of 5 mM. Data show an increase in the unfolding temperature of 2.9 ± 0.2°C in presence of 0.5 mM Mg^2+^. rh‐proNGF concentration is 10 μM with respect to the dimer. The curves are normalized according to the unfolding transition of the pro‐peptide domain. (b) Binding isotherm of the competition experiment: titration of 2 mM MgCl_2_ into 20 μM rh‐proNGF (dimer) and 40 μM ATP (cyan). The separate ATP/rh‐proNGF (violet) and MgCl_2_/ATP (blue) binding isotherms are also reported. DSF, differential scanning fluorimetry; ITC, isothermal titration calorimetry; NGF, nerve growth factor

We next investigated whether the ATP binding to rh‐proNGF might be fine‐tuned by Mg^2+^, known to play a physiological role in neurons (Mele et al., 2021) and to be physiologically relevant in ATP metabolism (Grauffel et al., 2019). DSF measurements in the presence of different ATP/Mg^2+^ ratios clearly show that at high concentrations of Mg^2+^ the effect of ATP on rh‐proNGF thermal stability becomes weaker (Figure 6a). Aiming at understanding the modulating role of Mg^2+^ ion, we also investigated the effects of Ca^2+^ and of Li^+^, known to have a lower affinity for ATP than Mg^2+^ (Wilson & Chin, 1991). The DSF behavior, in which equimolar—if compared to Mg^2+^—concentrations of Ca^2+^ or Li^+^ ions respectively were added to the system, was similar to that observed in the presence of Mg^2+^, albeit with a neatly reduced effect (Figure S7). This finding clearly points to the occurrence of both electrostatic and ion‐specific interactions modulating the Mg^2+^/ATP/rh‐proNGF interplay. The affinity of the interaction between Mg^2+^ and ATP (in the absence of rh‐proNGF) was measured by ITC (*K*_D_ = 21.8 μM) (Figure S8, Table S2) and is consistent with previously reported literature data (Wilson & Chin, 1991). Given the comparable affinities of ATP for rh‐proNGF (*K*_D_ = 39.7 μM) and Mg^2+^ (*K*_D_ = 21.8 μM), we next performed a competition experiment, titrating the pre‐formed ATP/rh‐proNGF complex with increasing concentrations of Mg^2+^. These data clearly show that Mg^2+^ and rh‐proNGF compete for ATP binding (Figure 6b). Furthermore, the absence of a second component in the titration curve indicates that a ternary ATP/rh‐proNGF/Mg^2+^ complex either does not form under these conditions, or it forms only at very low amounts.

**FIGURE 7:**
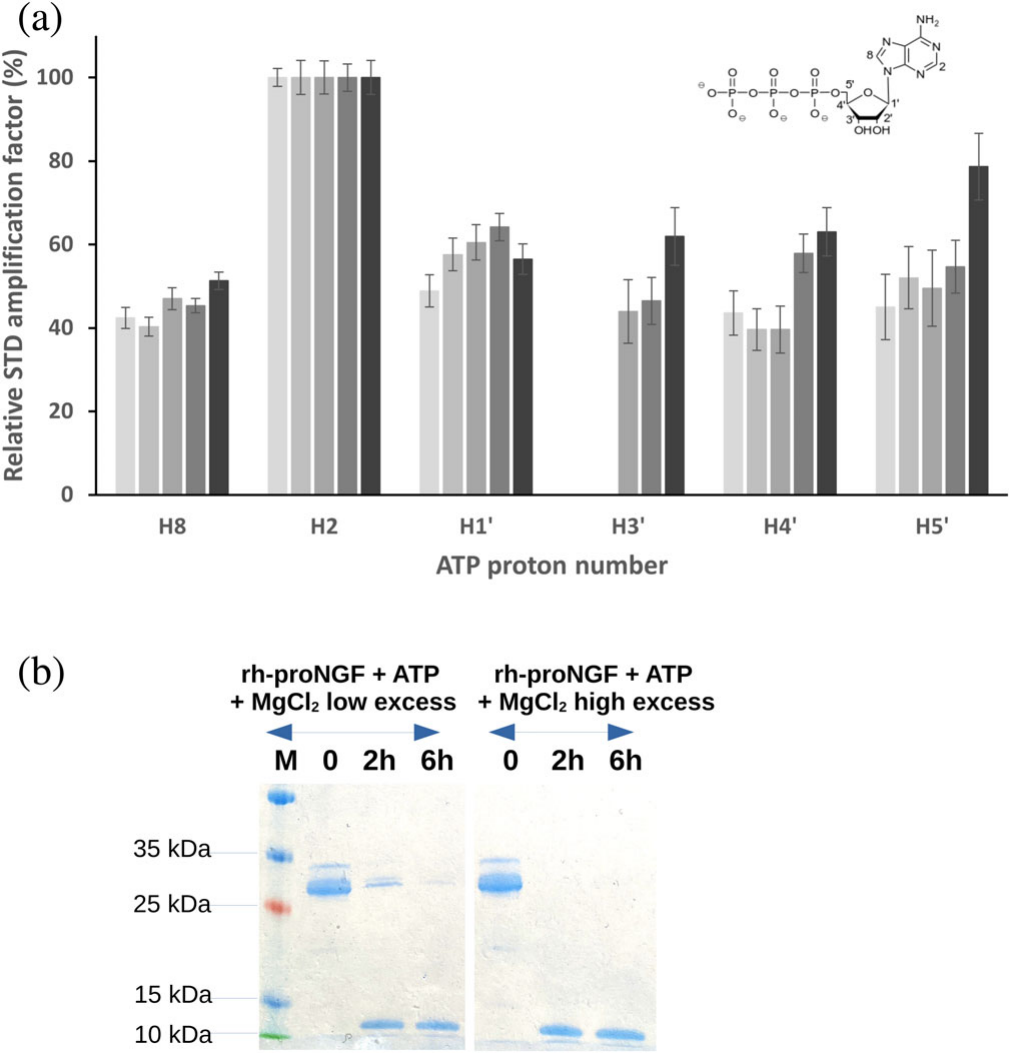
Effects of Mg^2+^ on ATP/rh‐proNGF interaction by ^1^H STD‐NMR (a) and limited proteolysis (b). (a) Relative degree of saturation of individual protons of ATP determined from the 1D ^1^H STD NMR spectrum at 100‐fold excess with respect to rh‐proNGF. Data with different amounts of MgCl_2_ are reported (from left to right columns, darker gray color moving from lower to higher Mg_2+_ concentrations): 0, 10, 100 μM, 1, 2 mM. STD values are normalized to the intensity of the signal with the largest STD effect. The absence of ATP H2’ proton STD amplification factors is due to the low intensity of signals in STD spectra and does not represent a loss of interaction. The inset shows the ATP molecule with the proton‐numbering scheme. (b) rh‐proNGF limited proteolysis with trypsin under controlled conditions. A 14% SDS‐PAGE with rh‐proNGF samples subjected to proteolysis with trypsin without and with ATP plus Mg^2+^. Used concentrations: dimeric rh‐proNGF: 8 μM; ATP: 240 μM; MgCl_2_ low excess (with respect to rh‐proNGF): 240 μM or MgCl_2_ high excess (with respect to rh‐proNGF): 1.6 mM. NGF, nerve growth factor; STD‐NMR, ^1^H saturation transfer difference NMR

**FIGURE 8:**
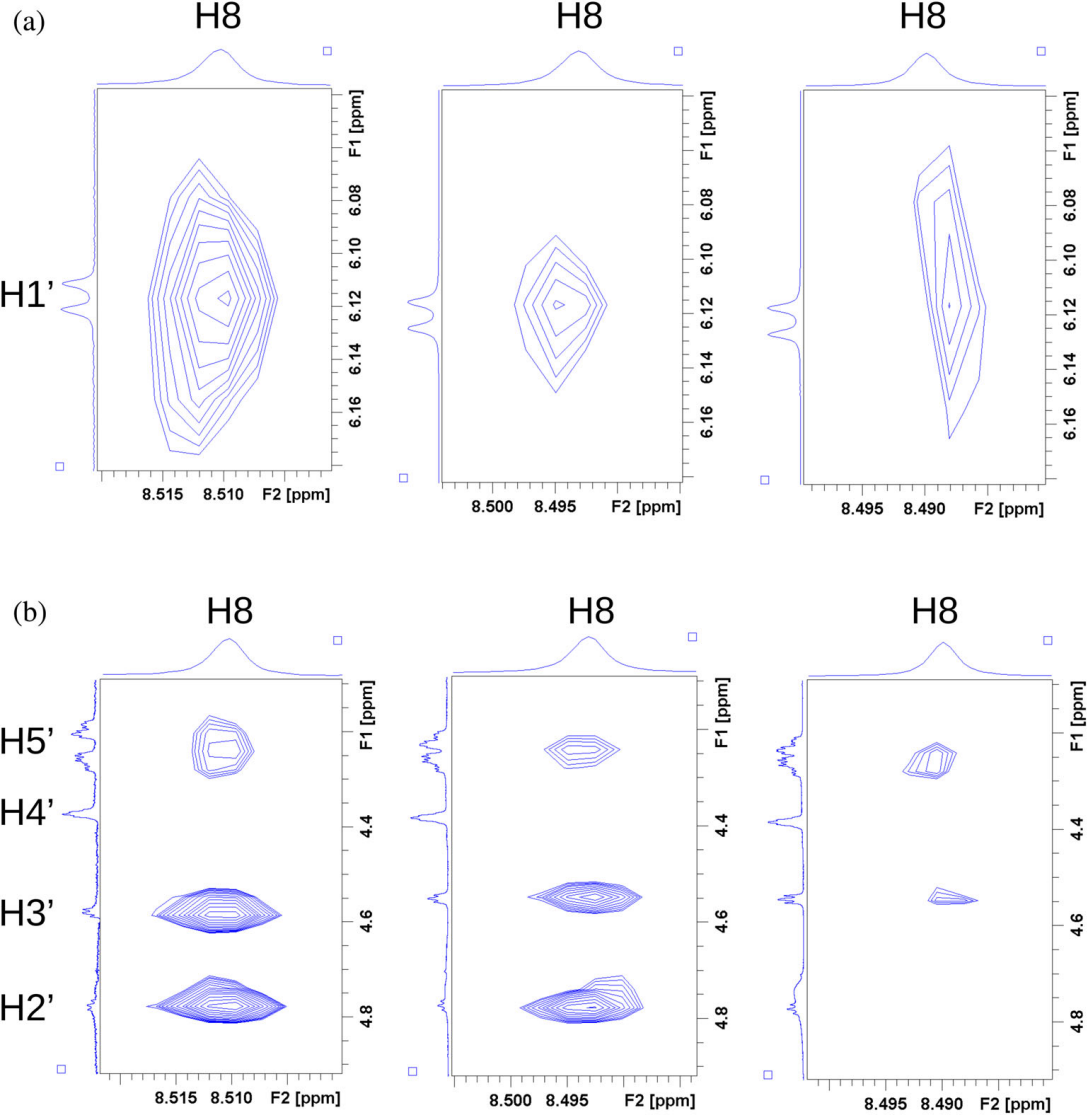
Selected expansions from trNOESY spectrum. The relevant NOEs in the trNOESY spectrum are shown in panels a) and b). The NOEs cross‐peaks have the same sign as the diagonal peaks (Figure S2). The compared spectra are those without MgCl_2_ (left figure in each panel), with equal amounts of ATP and MgCl_2_ (1 mM—central figure in each panel), and with a twofold excess of MgCl_2_ *versus* ATP (1 mM ATP, 2 mM MgCl_2_, right figure in each panel). At 2 mM Mg^2+^ concentration, the intensity of the ATP H8‐H1’ (a), H8‐H3’ (b), and H8‐H5’ (b) NOE cross‐peaks decreases, because of ATP weaker binding, as observed in ITC measurements. For the same reason, the H8‐H2’ NOE cross‐peak (b) disappears. ITC, isothermal titration calorimetry; trNOESY, transferred NOESY

^1^H STD‐NMR experiments with a constant ATP concentration and a variable amount of Mg^2+^ (Figure S9) showed that, moving towards higher [Mg^2+^], the STD amplification factor difference between the H2 and the other ATP protons becomes less pronounced than in the absence of Mg^2+^ (Figure 7a). This indicates that [Mg^2+^] affects the relative strength of the interaction of the adenine moiety compared to the sugar one. Changes in the NOEs intensities in the absence or presence of Mg^2+^ were also observed in the trNOESY experiment (Figure 8), indicating a different binding affinity in these conditions. This observation agrees with the ITC data, whereby at higher [Mg^2+^], the ion competes with rh‐proNGF for ATP, and thus the concentration of the ATP/rh‐proNGF complex is reduced. Further, competition of rh‐proNGF and Mg^2+^ for ATP was confirmed by limited proteolysis (Figure 7b). At low [Mg^2+^], the cleavage pattern of rh‐proNGF resembles that observed with ATP only; however, when there is a large excess of Mg^2+^ in addition to ATP, there is no protection from trypsin cleavage, thus confirming that ATP preferentially binds to Mg^2+^ over rh‐proNGF.

## 3 DISCUSSION

Recent evidence implicates a role for proNTs in regulating many biological functions, including in the nervous system (e.g., from apoptosis to pain), and an involvement of proNTs in disease states spanning neurodegeneration to inflammation. Despite recent studies, details of how the balance between mature and precursors NTs could affect their physiological functions in health and disease remains obscure (Bohmwald et al., 2022; Bothwell, 2016; Costa et al., 2018; Lewin & Nykjaer, 2014; Teng et al., 2010). Interestingly, proNTs are characterized by an intrinsically unstructured pro‐peptide (Covaceuszach et al., 2021), and this represents a possible evolutionary advantage that allows the protein to flexibly adapt and to quickly respond to a cell's needs in different physiological or pathological conditions (Toto et al., 2022).

In particular, many open questions pertaining to the regulation of NT signaling are related to the interplay between proteins and their binding partners, other than the receptors, especially small endogenous molecules (Paoletti & Lamba, 2021). Moreover, while the few available reports on this topic are focused on mature NTs, no insights are available on the binding of these small molecules to proNTs.

Here, we contribute to this gap in knowledge, by presenting the first biochemical/biophysical characterization of the binding of a small endogenous molecule to a neurotrophin precursor, namely ATP binding to rh‐proNGF. Our studies add to recent findings on the role of ATP as a protein binder beyond its energy functions (Nishizawa et al., 2021; Ou et al., 2021; Song, 2021). While it is out of the scope of our report to dissect the ultimate biological and cellular physiological significance of the rh‐proNGF/ATP/Mg^2+^ interaction, our results highlight the importance of small endogenous ligand interactions with IUDs in the context of cell signaling. Indeed, here we show that ATP induces changes in the conformation of the IUD pro‐peptide, suggesting a plastic adaptation of proNGF to different cellular environments. Furthermore, this interaction is tuned through active competition with Mg^2+^.

Overall, our experiments show that under thermodynamic equilibrium conditions, (i) the ATP/Mg^2+^ and ATP/rh‐proNGF moieties compete, (ii) that a ternary rh‐proNGF/ATP/Mg^2+^ complex does not form, and (iii) that ATP binding to rh‐proNGF can be modulated by the relative concentration of the three components (rh‐proNGF, ATP and Mg^2+^). This suggests an interplay of an unspecific electrostatic component to ATP/rh‐proNGF binding and a likely direct interaction—a very specific one—of ATPs negatively charged phosphate groups with positively charged protein residues (arginines, lysines, and histidines) (Hu et al., 2022). Interestingly, the pro‐peptide domain of rh‐proNGF is characterized by a higher proportion of positively charged residues, compared to the mature NGF domain (Figure 5a). This observation suggests the pro‐peptide domain may effectively be engaged in ATP interactions and may explain its higher binding affinity for rh‐proNGF compared to mature rhNGF. Recently, computer simulations pinpointed that the interplay between electrostatic attractions and favorable free energies of hydration creates distinct stable switch‐like transition states for polyampholytic intrinsically disordered regions (Zeng et al., 2022). It has also been shown that the charge distribution in IUDs affects their size and shape (Bianchi et al., 2022) as well as that arginines are involved in ATP modulation of liquid–liquid phase separation of the IUD protein TDP‐43 (Dang et al., 2021).

Given the different effects of ATP on receptor binding, it is tempting to speculate that ATP binding to proNGF might be connected to the modulation of proNGF signaling activity in different cellular contexts. As we have shown, the interplay between ATP, Mg^2+^, and proNGF is tuned by their abundance and relative stoichiometry. All three are key players in the cell's metabolism and their relative abundance in the cell is tightly regulated in vivo.

In physiological conditions, the extracellular ATP concentration [eATP] is in the nM range, that is, rather low if compared to the mM [ATP] found in the cytoplasm (Trautmann, 2009). However, the local pericellular [ATP] could reach the high μM range (Gordon, 1986). The signaling role of eATP is widely recognized, as well as the involvement of specific purinergic receptors (Burnstock, 2014). It is widely accepted that in damaged or dying cells, ATP is released in the extracellular space and its concentration can easily reach μM levels (Volonté et al., 2003). This is for example the case of an ischemic or hypoxic episode (Neary et al., 1996) as well as in trauma (Burnstock, 2008) and in other disorders of the CNS (Burnstock, 2008; Burnstock, 2016). In such circumstances, astrocytes and neurons would be exposed to high concentrations of these molecules. Indeed, in these conditions, nucleotides could act in synergy with growth factors to start a repair mechanism (Neary et al., 1996).

As far as [Mg^2+^] is concerned, it is well known that it is found at mM extracellular concentration in vivo (Yamanaka et al., 2019). Furthermore, Mg^2+^ has a neuroprotective effect in the CNS and the decline of free Mg^2+^ in the brain has been related to many pathological conditions, including ischemia, neurodegeneration, aging, trauma (Mele et al., 2021; Trapani et al., 2011). Mg^2+^ deprivation has been also proven to be followed by oxidative stress and subsequent induction of apoptosis (Trapani et al., 2011). Alzheimer's Disease (AD) patients show a lower [Mg^2+^] in the brain and in the cerebrospinal fluid, and this correlates with the severity of the disease (Yamanaka et al., 2019). [Mg^2+^] levels are also linked to the inflammation cascade (Pelczyńska et al., 2022).

Interestingly, the concentration of proNGF was proven as well to be tightly connected to the physiological‐to‐pathological transition. In physiological conditions, proNGF is processed extracellularly to mature NGF which ultimately exerts its physiological trophic functions (Pentz et al., 2021). However, it has been reported that in pathological conditions, such as those found in Alzheimer's Disease, the NGF metabolic pathway is impaired, with a reduction in proNGF processing and a concomitant increased NGF degradation (Pentz et al., 2021) and increased proNGF levels found in AD correlate with the severity of the disease (Counts & Mufson, 2005). The absence of proNGF degradation in these conditions remains obscure, although a role of the pro‐peptide in this process has been proposed (Pentz et al., 2021).

**FIGURE 9:**
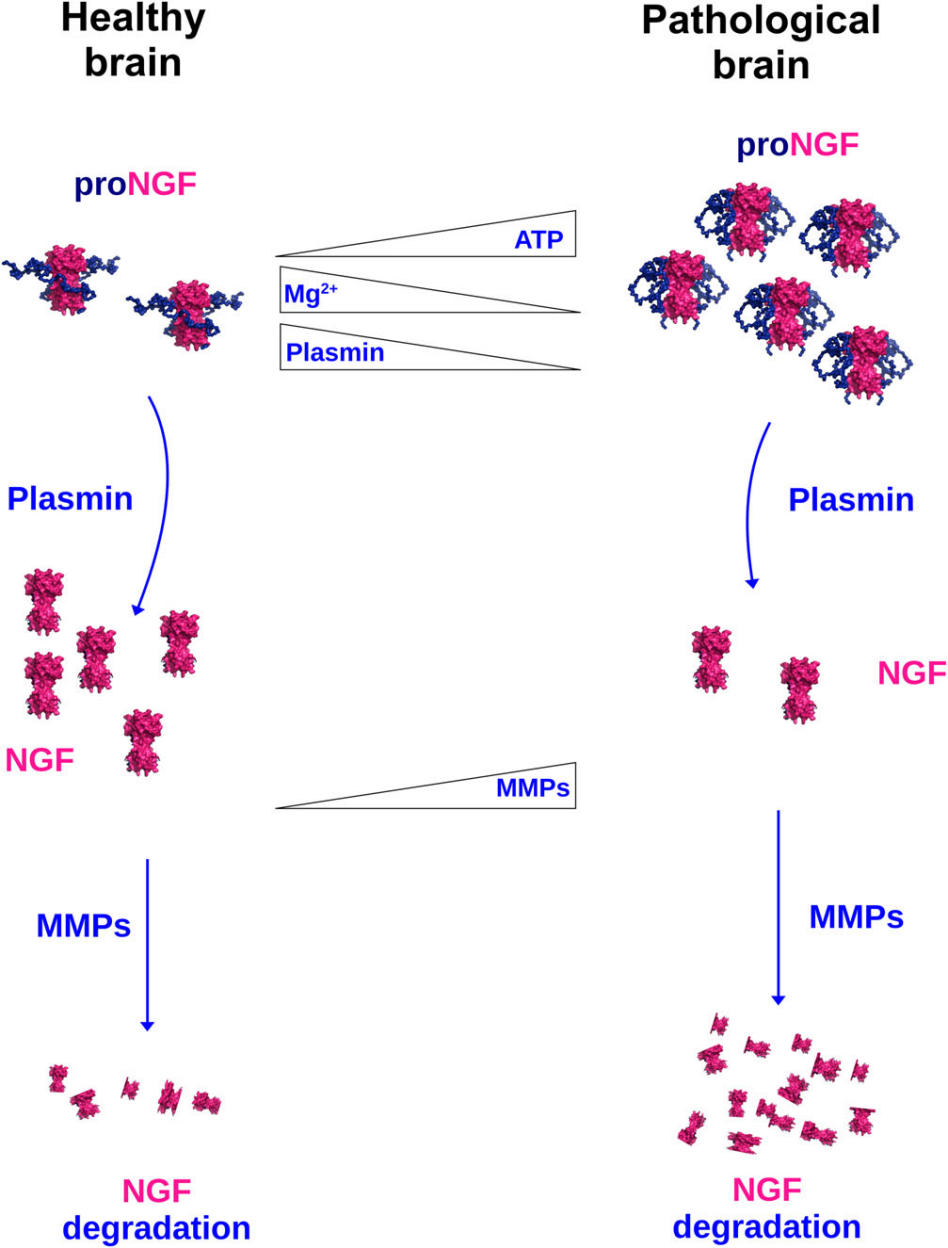
Schematic representation of the proposed functional role of ATP binding to proNGF in health and disease conditions. A schematic representation of the cascade of events taking place in health (left side) and disease (right side) are reported in cartoon mode. The molecules involved are drawn and/or labeled. The triangles in the central part of the figure represent the change in the concentration of the molecules when comparing the health or disease conditions. Briefly, in healthy brain conditions (left side), proNGF is processed by plasmin to mature NGF in the extracellular space. NGF then exerts its biological function and undergoes its physiological degradation levels by MMPs (Pentz et al., 2021). In the pathological brain conditions (right side), the lower Mg^2+^ concentration and a higher ATP concentration together with a higher proNGF amount, promote ATP binding to proNGF and a consequent conformational rearrangement in the pro‐peptide of proNGF. At the same time, the reduced plasmin concentration causes a reduction in proNGF processing and thus an accumulation of proNGF and reduced NGF amounts (Pentz et al., 2021). The concomitant increased MMPs concentration causes an increase in NGF degradation, while proNGF is protected from the degradation in its ATP‐bound form. NGF, nerve growth factor, MMPs, matrix metalloproteinases

Our data propose a possible explanation for this observation. Indeed, in conditions of low [Mg^2+^], ATP binds proNGF with a μM *K*_D_, while at higher [Mg^2+^] it would act as a competitor for ATP with respect to proNGF. As we discussed above, the occurrence of an increased [eATP], decreased [Mg^2+^] and increased proNGF amounts happens in vivo in different pathologies. The modulating effects of ions on ATP binding to IUD proteins as well as to flexible regions of folded proteins have been recently reported (Nishizawa et al., 2021). In line with this, ATP binding to proNGF in conditions of cell stress induces a conformational change in the pro‐peptide of proNGF that in turn affects the processing to mature NGF. Our proposed working idea is illustrated in Figure 9, where the physiological *versus* pathological proNGF metabolism conditions are schematically depicted, together with the changes in [eATP] and [Mg^2+^]. ATP binding to proNGF in pathological conditions would induce an alteration of NGF/proNGF homeostasis and thus their relative bioavailability, affecting their interaction with the receptors. This is consistent with a role for the pro‐domain of NGF in regulating signaling and function and also well agrees with the reported cross‐talk between the purinergic receptors system and the neurotrophins’ receptors system in mediating cell death (Cavaliere et al., 2002).

We believe that this study paves the way for further investigations on the role of ATP and other small ligands as conformational allosteric modulators of the bioactivity of NGF, proNGF, and possibly also the other neurotrophic factors and their precursors in health and disease (Miras‐Portugal et al., 2016).

## 4 MATERIALS AND METHODS

Extended materials and methods can be found in Appendix S1.

### 4.1 Expression, refolding, and purification of recombinant human proNGF

Recombinant human proNGF (rh‐proNGF) was expressed in Rosetta (DE3) *Escherichia Coli* cells. Solubilization and refolding of rh‐proNGF from inclusion bodies were carried out according to published protocols (Paoletti et al., 2016; Rattenholl et al., 2001).

### 4.2 Nuclear Magnetic Resonance (NMR)

The ^1^H STD and the tr‐NOESY experiments were recorded on a Bruker Avance Neo 600 MHz spectrometer using a cryoprobe at 30°C on samples containing 10 μM unlabeled rh‐proNGF in 50 mM Tris‐*d*_11_ and 50 mM NaCl in D_2_O, pD 7.3 buffer. The spectra were recorded at an ATP/rh‐proNGF ratio of 100:1, that is a protein concentration of 10 μM (with respect to the dimer) and 1 mM ATP. To investigate the effect of the cation, MgCl_2_ was added to the samples, at the following concentrations: 0, 10, 100 μM, and 1, 2 mM.

The ^1^H STD ligand epitope mapping experiments (Mayer & Meyer, 2001) were performed under quantitative conditions, considering the non‐uniform relaxation properties of ATP. Errors in the STD amplification factor were estimated according to the formula: STD amplification factor absolute error = STD amplification factor × ((*N*_STD_/*I*_STD_)^2^ + (*N*_REF_/*I*_REF_)^2^)^1/2^ (McCullough et al., 2012). *N*_STD_ and *N*_REF_ are noise levels in STD and reference spectra. *I*_STD_ and *I*_REF_ are signal intensities in STD and reference spectra.

The tr‐NOESY spectra were recorded with a 6578 Hz sweep width, 4096 data points in *t*_2_, 48 scans, 128 complex data points in *t*_1_, a mixing time of 350 ms, and a relaxation delay of 1.5 s.

Please find experimental details in Appendix S1.

### 4.3 Isothermal titration microcalorimetry

Isothermal titration microcalorimetry (ITC) experiments were carried out using an ITC 200 microcalorimeter (Malvern Panalytical, UK). rh‐proNGF was extensively dialyzed against 50 mM Hepes pH 7.2; 100 mM ATP and 1 M MgCl_2_.

ATP/rh‐proNGF titrations were performed at 30.0 ± 0.1°C with a stirring rate set to 750 rpm to ensure rapid mixing. Twenty injections of 2 μl of 2 mM ATP into a measurement cell filled with 200 μl of 20 μM dimeric rh‐proNGF solution, were performed at time intervals of 240 s.

Integration of the raw thermograms has been done with the program NITPIC (Keller et al., 2012).

Global fitting of the titration experiments by nonlinear least squares was performed to analyze the ATP/rh‐proNGF binding isotherms by means of SEDPHAT software (Zhao et al., 2015).

### 4.4 Differential scanning fluorimetry

Differential scanning fluorimetry (DSF) experiments were performed in triplicate using a CFX96 Touch Biorad real‐time PCR instrument (Bio‐Rad). rh‐proNGF (10 μM with respect to dimer) in 50 mM Hepes, 150 mM NaCl, pH 7.2, was pre‐incubated with ATP and/or MgCl_2_ or CaCl_2_ or LiCl for 30 min at 4°C before adding SYPRO orange dye (Sigma) at a final concentration of 90×. The fluorescence was measured as a function of increasing temperature in the 20–90°C range at the rate of 0.2°C/min (excitation wavelength: 470–505 nm; emission wavelength: 540–700 nm). Melting temperatures (*T*_m_) were obtained by fitting the sigmoidal melt curves to the Boltzmann equation (Ericsson et al., 2006).

### 4.5 Fourier transform ATR infrared spectroscopy

Fourier transform infrared (FT‐IR) spectra were recorded on the spectrometer Bruker Vertex 80 equipped with the Golden Gate ATR. Please find experimental details in Appendix S1.

### 4.6 Small‐angle X‐ray scattering measures and data processing

Small angle X‐ray scattering (SAXS) experiments were conducted at the P12 beamline EMBL SAXS‐WAXS at PETRAIII/DESY (Blanchet et al., 2015) (Hamburg, Germany). Data were collected as 20 × 0.05 s exposures on a Pilatus 2 M (Dectris) detector, with a sample‐detector distance of 3.00 m and a wavelength of 1.24 Å. Six different concentrations in 50 mM Sodium Phosphate, 1 mM EDTA, pH 7.0, and 1 mM ATP (Table S2) were measured without detectable radiation damage effects by comparing the scattering curves of the collected frames.

After normalization to the intensity of the transmitted beam, data merging for each sample, subtraction of the scattering of the buffer, and the following processing were performed using PRIMUS (Konarev et al., 2003) as implemented in the ATSAS 2.8 suite (Franke et al., 2017). For details of *R*_g_ and *p*(*r*) calculations, rigid body modeling by CORAL (Petoukhov et al., 2012), and quantitative analysis of the size distribution of possible conformers by EOM (Bernadó et al., 2007) see Appendix S1.

### 4.7 Hydrogen‐deuterium exchange mass spectrometry

HDX‐MS experiments were performed by implementing an automated robot (LEAP Technologies) coupled to an Acquity M‐Class LC with HDX manager (Waters). Samples contained 10 μM (with respect to the dimer) rh‐proNGF, with or without 1 mM ATP, in 10 mM potassium phosphate, pH 7.0. A volume of 95 μl of deuterated buffer (10 mM potassium phosphate, pD 7.0) was added to 5 μl of protein‐containing solution, and the mixture was incubated at 4°C for 0.5, 2, and 30 min. Three replicate measurements were performed for each time point and condition. Data analysis was performed using PLGS (v3.0.2) and DynamX (v3.0.0) (Waters). A summary of the HDX‐MS data, as per recommended guidelines, is shown in Table S3.

### 4.8 Limited proteolysis

The non‐specific protease trypsin was used for the limited proteolysis experiments under controlled conditions. An amount of 16 μg of rh‐proNGF at the concentration of 8 μM (with respect to dimer) in 50 mM Hepes, pH 7.2, were proteolytically digested by trypsin (Promega Corporation, Madison, USA) at 4°C. The reaction was started by adding trypsin at the ratio of 1:300 (enzyme:substrate).

### 4.9 Surface plasmon resonance

Experiments were performed with a Biacore T200 equipment (Cytiva Life Sciences), equipped with Biacore T200 Control software. Please find experimental details in Appendix S1.

## AUTHOR CONTRIBUTIONS

**Francesca Paoletti:** Conceptualization (lead); formal analysis (lead); investigation (lead); validation (lead); writing – original draft (lead). **Sonia Covaceuszach:** Conceptualization (equal); formal analysis (equal); investigation (equal); validation (equal); writing – review and editing (equal). **Alberto Cassetta:** Conceptualization (supporting); formal analysis (supporting); investigation (supporting); validation (supporting). **Antonio Calabrese:** Formal analysis (supporting); investigation (supporting); validation (supporting); writing – review and editing (supporting). **Urban Novak:** Investigation (supporting); validation (supporting). **Petr Konarev:** Formal analysis (supporting); validation (supporting). **Jože Grdadolnik:** Formal analysis (supporting); funding acquisition (equal); validation (supporting). **Doriano Lamba:** Conceptualization (equal); formal analysis (equal); validation (equal); writing – review and editing (equal). **Simona Golič Grdadolnik:** Conceptualization (lead); formal analysis (lead); funding acquisition (equal); investigation (lead); validation (lead); writing – review and editing (equal).

## CONFLICT OF INTEREST

The authors declare no conflict of interest.

## Supporting information


**Appendix S1:** Supporting Information
