# Supplementary material for: Distinct conformational changes occur within the intrinsically unstructured pro‐domain of pro‐Nerve Growth Factor in the presence of ATP and Mg2+
Source: Protein Sci. 2023 Feb 1;32(2):e4563. doi: 10.1002/pro.4563 (PMC9878617; doi:10.1002/pro.4563)
Supplement: Supplementary file 1 — Appendix S1: Supporting Information [file PRO-32-e4563-s001.docx]

**Supplementary Material for:**

**Distinct conformational changes occur within the intrinsically unstructured pro-domain of pro-Nerve Growth Factor in the presence of ATP and Mg^2+^**

Francesca Paoletti*^[1][a]^, Sonia Covaceuszach^[1][b]^, Alberto Cassetta^[b]^, Antonio N. Calabrese^[c]^, Urban Novak^[a]^, Petr Konarev^[d]^, Jože Grdadolnik^[a]^, Doriano Lamba^[b] [e]^ and Simona Golič Grdadolnik*^[a]^

^[a]^ Laboratory for Molecular Structural Dynamics, Theory Department, National Institute of Chemistry, Hajdrihova 19, SI-1001 Ljubljana, Slovenia

^[b]^ Institute of Crystallography - C.N.R. - Trieste Outstation, Area Science Park – Basovizza, S.S.14 - Km. 163.5, I-34149 Trieste, Italy

^[c]^ School of Molecular and Cellular Biology, Astbury Centre for Structural Molecular Biology

University of Leeds, Leeds, LS2 9JT, UK

^[d]^ A.V. Shubnikov Institute of Crystallography of Federal Scientific Research Centre "Crystallography and Photonics", Russian Academy of Sciences, Leninsky pr. 59, 119333 Moscow, Russia

^[e]^ Interuniversity Consortium “Biostructures and Biosystems National Institute”, Viale delle Medaglie d'Oro 305, I-00136 Roma, Italy

* Dr. Francesca Paoletti, Prof. Simona Golič Grdadolnik

Email: simona.grdadolnik@ki.si; francesca.paoletti@ki.si

^[1]^ Equal contribution

Table of Contents

Materials and Methods 5

Expression, refolding and purification of recombinant human proNGF (rh-proNGF) 5

Nuclear Magnetic Resonance (NMR) 5

Isothermal Titration microCalorimetry (ITC) 6

Differential Scanning Fluorimetry (DSF) 8

Fourier Transform Infrared Spectroscopy (FT-IR) 8

Small-Angle X-ray Scattering (SAXS) measures and data processing 8

Hydrogen-Deuterium Exchange Mass Spectrometry (HDX-MS) 10

Limited proteolysis 11

Surface Plasmon Resonance (SPR) 12

References 14

Figures and Tables 16

Figure S1 – Interaction of rh-proNGF with ATP by 1D ^1^H Saturation Transfer Difference NMR experiment (^1^H STD-NMR) 16

Figure S2 – Selected expansion from transferred NOESY (trNOESY) spectrum 18

Figure S3 – Fourier Transform Infrared Spectroscopy (FT-IR) of rh-proNGF in absence and in presence of ATP 19

Figure S4 – HDX-MS data of rh-proNGF in the absence and presence of ATP 20

Figure S5 – rh-proNGF limited proteolysis with trypsin under controlled conditions 22

Figure S6 – Single Cycle Kinetics SPR sensorgrams in absence and in presence of different amounts of ATP 23

Figure S7 – DSF demonstrates that cations modulate ATP interaction with rh-proNGF 25

Figure S8 – Interaction of MgCl_2_ with ATP by ITC 26

Figure S9 – Interaction of rh-proNGF with ATP by 1D ^1^H STD-NMR experiment 27

Table S1 – SAXS structural parameters 28

Table S2 – Best fit valued obtained by global fitting of two independent ITC titrations 30

Table S3 – HDX Data Summary Table 31

**Materials and Methods**

**Expression, refolding and purification of recombinant human proNGF (rh-proNGF)**

Recombinant human proNGF (rh-proNGF) was expressed in Rosetta (DE3) *E. Coli* cells, transformed with the pET22b plasmid containing the cDNA of human proNGF without secretory periplasmic signal ^1^. Solubilization and refolding of rh-proNGF from inclusion bodies was carried out according to published protocols ^2,3^.

**Nuclear Magnetic Resonance (NMR)**

The ^1^H STD experiments were recorded on a Bruker Avance Neo 600 MHz spectrometer using a cryoprobe at 30°C on samples containing 10 µM unlabeled rh-proNGF in 50 mM Tris-*d*_11_ and 50 mM NaCl in D_2_O, pD 7.3 buffer. The residual water was suppressed using excitation sculpting with 5 ms selective pulse. T_1_ρ filter of 30 ms was used to eliminate the background protein resonances. The STD spectra were recorded at an ATP/rh-proNGF ratio of 100:1, that is a protein concentration of 10 µM (with respect to the dimer) and 1mM ATP. To investigate the effect of the cation, MgCl_2_ was added to the samples, at the following concentrations: 0, 10 µM, 100 µM, 1 mM, 2 mM.

The ^1^H STD ligand epitope mapping experiments ^4^ were performed under quantitative conditions, considering the non-uniform relaxation properties of ATP. The inversion recovery T_1_ experiments showed that the ^1^H T_1_ relaxation times of ATP range from 3.9 s in the adenine moiety to 0.43 s in the sugar moiety. Therefore, STD amplification factors were determined with a short saturation delay of 0.21 s to avoid the effect of the longitudinal relaxation rate on the signal intensities ^5^. Spectra were recorded with a 6578 Hz spectral width, 16384 data points, a relaxation delay of 5 s and 4500-10600 scans. Selective saturation was achieved by a train of 50 ms long Gauss-shaped pulses separated by 1 ms delay. The on-resonance selective saturation of rh-proNGF was applied at 0.32 ppm at transmitter offset referenced to 4.64 ppm. The off-resonance irradiation was applied at 30 ppm for the reference spectrum. Subtraction of the on and off-resonance spectra was performed internally via phase cycling. Spectra were zero-filled twice and apodized by an exponential line-broadening function of 3 Hz. The STD amplification factor for the H2 proton was corrected based on the residual signal observed for the H2 proton in the STD control experiment on a sample containing ATP without protein (Fig S1). Errors in the STD amplification factor were estimated according to the formula: STD amplification factor absolute error = STD amplification factor × ((N_STD_/I_STD_)^2^ + (N_REF_/I_REF_)^2^)^1/2^ ^6^. N_STD_ and N_REF_ are noise levels in STD and reference spectra. I_STD_ and I_REF_ are signal intensities in STD and reference spectra.

The tr-NOESY spectra were recorded on a Bruker Avance Neo 600 MHz spectrometer using a cryoprobe at 30°C with 6578 Hz sweep width, 4096 data points in t_2_, 48 scans, 128 complex data points in t_1_, mixing time of 350 ms and a relaxation delay of 1.5 s. Spectra were zero-filled twice and apodized with a squared sine bell function shifted by π/2 in both dimensions.

**Isothermal Titration microCalorimetry (ITC)**

ITC experiments were carried out using an ITC 200 microcalorimeter (Malvern Panalytical, UK). rh-proNGF was extensively dialyzed against 50mM Hepes pH 7.2; 100 mM ATP and 1 M MgCl_2_ stock solutions were prepared by dissolving ATP or MgCl_2_ into a proper volume of the dialysis buffer. The concentration of rh-proNGF solutions were determined using a NanoDrop LITE Spectrophotometer (Thermo Fisher Scientific, US) prior to ITC measurements. Both rh-proNGF, ATP and MgCl_2_ solutions were centrifuged for 5 min at 13000 rpm and then degassed. All the titrations were done at least in duplicate.

ATP/rh-proNGF titrations were performed at 30.0°±0.1°C with a stirring rate set to 750rpm to ensure rapid mixing. 20 injections of 2 μL of 2 mM ATP into a measurement cell filled with 200 μL of 20 μM dimeric rh-proNGF solution, were performed at time intervals of 240 s. The first injection was of 0.2 μL only and then excluded in the analysis. A further set of titrations was performed following the same protocol but loading 5 mM ATP into the titration syringe. Negative control titrations to evaluate ATP dilution heats were performed according to the previous protocol by injecting 2 mM ATP the cell filled with the buffer solution only.

MgCl_2_ titrations into ATP were performed at 30.0°±0.1°C with a stirring rate set to 750rpm. A set of 40 injections of 1 μL of 20 mM MgCl_2_ (0.2 μL the first injection, excluded in the analysis) into the measurement cell filled with 200 μL of 2 mM ATP, were performed at time intervals of 240 s. A further set of titrations were done by injecting 20 mM MgCl_2_ into the buffer only, in order to establish the absence of any specific interaction of divalent ions with buffer components and to evaluate the entity of MgCl_2_ dilution heats.

To perform the competition experiments, a first set of 20 injections of 2 μL of 2 mM MgCl_2_ into the 200 μL measurement cell containing 20 μM dimeric rh-proNGF and 40 μM ATP were done at time interval of 240 s. The first set of titrations was followed by a further set of 20 injections into the same cell. The first injections of both set of measurements were of 0.2 μL in volume and then excluded in the analysis. The control experiment of the titration of MgCl_2_ into dimeric rh-proNGF did show no binding (data not shown).

Integration of the raw thermograms has been done with the program NITPIC ^7^ that allowed for a precise and unbiased extraction of the heats from the experimental curves. NITPIC has been used also to join the two consecutive sets of titrations in competition measurements.

Global fitting of two out of three ATP/rh-proNGF titration experiments by nonlinear least squares was performed to analyze the ATP/rh-proNGF binding isotherms by means of SEDPHAT software ^8^. A model describing one binding sites on each protomer of rh-proNGF was enforced in fitting procedures. The binding sites on the two protomers were assumed to be symmetric, as for mature rhNGF ^9^.

Global fitting of two MgCl_2_/ATP titrations were also performed in SEDPHAT in order to determine the binding parameters to be used in the following competition experiment analysis. Competition data were fitted in SEDPHAT by imposing log(Ka) and 𝛥H of both ATP/rh-proNGF and MgCl_2_/ATP systems as obtained by the independent analysis previously described and letting only the baseline and the incompetent fraction for ATP and rh-proNGF to be refined (evaluated as 5% and 25% for rh-proNGF and ATP, respectively).

**Differential Scanning Fluorimetry (DSF)**

DSF experiments were performed in triplicate using a CFX96 Touch Biorad real-time PCR instrument (Bio-Rad). rh-proNGF (10µM with respect to dimer) in 50 mM Hepes, 150 mM NaCl, pH 7.2, was pre-incubated with ATP and/or MgCl_2_ or CaCl_2_ or LiCl for 30 minutes at 4°C before adding SYPRO orange dye (Sigma) at a final concentration of 90x. The fluorescence was measured as a function of increasing temperature in the 20°- 90° C range at the rate of 0.2°C/min (excitation wavelength: 470–505 nm; emission wavelength: 540–700 nm). Melting temperatures (Tm) were obtained by fitting the sigmoidal melt curves to the Boltzmann equation^10^.

**Fourier Transform ATR Infrared Spectroscopy (ATR-IR)**

Infrared Spectra were recorded on the spectrometer Bruker Vertex 80 equipped with the Golden Gate ATR. Typically, 64 spectra were recorded at 25°C with a resolution of 4 cm^-1^. The spectra were processed using the OPUS software. rh-proNGF at 0.15 mM concentration (with respect to the dimer) in 50 mM Hepes, pH 7.2 was used. The visibility of the amide I and amide II bands was achieved by subtracting the original protein solution spectra with the spectrum of the Hepes buffer alone.

**Small-Angle X-ray Scattering (SAXS) measures and data processing**

SAXS experiments were conducted at the P12 beamline EMBL SAXS-WAXS at PETRAIII/DESY^11^ (Hamburg, Germany). Data were collected as 20 x 0.05 s exposures on a Pilatus 2M (Dectris) detector, with sample-detector distance 3.00 m and a wavelength 1.24 Å. Six different concentrations in 50 mM Sodium Phosphate, 1mM EDTA, pH 7.0, 1mM ATP (the range is reported in Table S1) were measured without detectable radiation damage effects comparing the scattering curves of the collected frames.

After normalization to the intensity of the transmitted beam, data merging for each sample, subtraction of the scattering of the buffer and the following processing were performed using PRIMUS ^12^ as implemented in the ATSAS 2.8 suite^13^.

The forward scattering I(0) and the radius of gyration R_g_ of the solute proteins were calculated by the Guinier approximation^14^ at very small angles (s < 1.3/Rg), assuming that the intensity is represented as I(s) = I(0)* exp(-1/3(R_g_*s)^2^), and from the entire scattering pattern by the program GNOM ^15^. GNOM was employed also to evaluate the pair distance distribution function p(r) and the maximum sizes D_max_ of the particles in solution. Molecular weights (MM) were computed comparing the calculated I(0) values with that of the standard solution of bovine serum albumin (MM 66 kDa). The excluded volume of the hydrated protein molecule (V_p_) was obtained using the Porod approximation ^16^:

$V_{p}=\frac{2\pi^{2}I\left( 0 \right)}{\int I_{exp}\left( s \right)s^{2}ds}$ .

$V_{p}=\frac{2\pi^{2}I\left( 0 \right)}{\int I_{\exp}\left( s \right)s^{2}\mathrm{ds}}$The *AMBIMETER* tool^17^ was employed to estimate the ambiguity of 3D reconstruction from a library of normalized scattering profiles from relatively simple shapes. The resulting ambiguity scores (0.9031 and 1.079, without and with ATP, respectively) suggest that in both conditions the 3D reconstruction is potentially unique. The MM calculated from the forward intensity at zero angle I(0) and the excluded Porod Volume V_p_ confirm the dimeric assembly at all the concentration tested.

In addition, rigid-body modeling using CORAL ^18^ was employed to model clash-free configurations of the pro-peptide domains of the protomers, (113 residues at the N-terminals and 4 residues at the C-terminals), keeping fixed the obtained high resolution 3D model of rhNGF^19^ and imposing a P2 symmetry.

The ensemble optimization method (EOM) ^20^ was employed to quantitatively analyse the size distribution of possible conformers. This method allows the co-existence of multiple conformations in solution, whose average scattering fits the experimental data. After the generation of an initial random pool of 10000 conformers, the the obtained high resolution 3D model of rhNGF^19^ was used as rigid body, imposing P2 symmetry and the modelled missing regions were allowed to have native-like conformations. CRYSOL was used to compute the theoretical scattering for each generated model and a genetic algorithm (GAJOE) was employed to select an ensemble of conformations whose combination best fitted the experimental data.

**Hydrogen-Deuterium Exchange Mass Spectrometry (HDX-MS)**

HDX experiments were performed by implementing an automated robot (LEAP Technologies) coupled to an Acquity M-Class LC with HDX manager (Waters). Samples contained 10 µM (respect to the dimer) rh-proNGF, with or without 1 mM ATP, in 10 mM potassium phosphate, pH 7.0. 95 μL of deuterated buffer (10 mM potassium phosphate, pD 7.0) was added to 5 μL of protein-containing solution, and the mixture was incubated at 4 °C for 0.5, 2, 30 min. Three replicate measurements were performed for each time point and condition. 100 μL of quench buffer (10 mM potassium phosphate, 0.05 % DDM, pH 2.2) was added to 50 μL of the labelling reaction and 50 μL of the quenched sample was injected onto immobilised pepsin and aspergillopepsin columns (Affipro) connected in series (20 °C). A VanGuard Pre-column [Acquity UPLC BEH C18 (1.7 μm, 2.1 mm × 5 mm, Waters)] was used to trap the resultant peptides for 3 min. A C18 column (75 μm × 150 mm, Waters, UK) was used to separate the peptides, employing a gradient elution of 0–40% (v/v) acetonitrile (0.1% v/v formic acid) in H_2_O (0.3% v/v formic acid) over 7 min at 40 μL min^−1^. The eluate from the column was infused into a Synapt G2Si mass spectrometer (Waters) that was operated in HDMS^E^ mode. The peptides were separated by ion mobility prior to CID fragmentation in the transfer cell, to enable peptide identification. Deuterium uptake was quantified at the peptide level. Data analysis was performed using PLGS (v3.0.2) and DynamX (v3.0.0) (Waters). Search parameters in PLGS were: peptide and fragment tolerances = automatic, min fragment ion matches = 1, digest reagent = non-specific, false discovery rate = 4. Restrictions for peptides in DynamX were: minimum intensity = 1000, minimum products per amino acid = 0.3, max sequence length = 25, max ppm error = 5, file threshold = 3. Peptides with statistically significant increases/decreases in deuterium uptake were identified using the software Deuteros^21^. Deuteros was also used to prepare Wood’s plots. The raw HDX-MS data have been deposited to the ProteomeXchange Consortium via the PRIDE partner repository with the dataset identifier PXD035625.  A summary of the HDX-MS data, as per recommended guidelines, is shown in Table S3.

**Limited proteolysis**

The non-specific protease trypsin was used for the limited proteolysis experiments under controlled conditions. Trypsin, as previously reported, cleaves the more accessible pro-peptide, and leaves NGF undigested under controlled conditions^22^. 16 μg of rh-proNGF at the concentration of 8 µM (with respect to dimer) in 50 mM Hepes, pH 7.2, were proteolytically digested by trypsin (Promega Corporation, Madison, USA) at 4°C. The reaction was started adding trypsin at the ratio of 1:300 (enzyme: substrate). 5 μl of the reaction mixtures were taken at time 0, and after 2 and 6 hours. For each sample, the reaction was blocked by addition of Laemmli sample buffer and boiling for 10 minutes. All the samples were analyzed by 14% SDS-PAGE. The experiment was repeated three times on independent samples.

**Surface Plasmon Resonance (SPR)**

Experiments were performed with a Biacore T200 equipment (Cytiva Life Sciences), equipped with Biacore T200 Control software. The amine coupling reaction was performed, according to manufacturer’s instructions (Cytiva Life Sciences) on CM5 chips.

TrkA (R&D, TrkA Fc Chimera, 175-TK-050), p75^NTR^ (R&D, R/TNFRSF16 Fc Chimera, 367-NR-050) and sortilin (R&D, recombinant human sortilin, 3154-ST) receptors extracellular domains were used as ligands, dissolved in 10 mM Sodium Acetate pH 5 at 20 µg/mL and immobilized at a 300 RU surface concentration on CM5 chip.

The IUD nature of the pro-peptide contributes to an increasing complication in SPR measurements, due to the aspecific interaction of the analyte to the chip dextran surface. To reduce unwanted binding, a high ionic strength in the running buffer is required, as well as a careful optimization of the regeneration conditions to avoid surface modifications. In analogy to what previously reported for SPR on rh-proNGF ^23^ we opted for (NH_4_)_2_SO_4_ as the salt to be used in the running buffer. This is preferred to NaCl because of the lower interaction with ATP and because it was verified that it can be used at lower concentration than NaCl to reduce unwanted binding. The running buffer was thus 20 mM Hepes, 100 mM (NH_4_)_2_SO_4_, 0.05% Polysorbate 20, pH 7.2.

After a careful set of optimization experiments, the best conditions for the chip regeneration were found. Due to the harsh condition needed to regenerate the surface, the strategy was selected to measure the kinetics as a Single Cycle Kinetic (SCK) experiment, which allows regeneration to take place only at the end of the kinetic series of injections, and thus represents a lower stress for the ligands bound on the surface. Data evaluation was difficult due to the very slow dissociation of rh-proNGF from the surface, which contributes to an estimation of the *K*_D_ at the limits of the instrument sensitivity. A high flow rate (70 μl/min) was used over the experiments, to reduce mass transport limitations.

For the SCK the following conditions were employed. Contact time: 300 s; dissociation time: 1200 s. Regeneration: five pulses of 50 mM NaOH, 30 s contact time, 120 s of stabilization time. For the SCK, 5 dilutions of rh-proNGF were measured in the 1.5 nM-15 nM concentration range, with respect to the dimer. Every SCK was repeated at least 5 times. Data analysis was carried out using Biacore T200 Evaluation Software.

**References**

1. Covaceuszach S, Capsoni S, Ugolini G, Spirito F, Vignone D, Cattaneo A (2009) Development of a non invasive NGF-based therapy for Alzheimer’s disease. Curr Alzheimer Res 6:158–170.

2. Rattenholl A, Lilie H, Grossmann A, Stern A, Schwarz E, Rudolph R (2001) The pro-sequence facilitates folding of human nerve growth factor from Escherichia coli inclusion bodies. Eur. J. Biochem. 268:3296–3303.

3. Paoletti F, de Chiara C, Kelly G, Covaceuszach S, Malerba F, Yan R, Lamba D, Cattaneo A, Pastore A (2016) Conformational Rigidity within Plasticity Promotes Differential Target Recognition of Nerve Growth Factor. Front Mol Biosci 3:83.

4. Mayer M, Meyer B (2001) Group epitope mapping by saturation transfer difference NMR to identify segments of a ligand in direct contact with a protein receptor. J. Am. Chem. Soc. 123:6108–6117.

5. Yan J, Kline AD, Mo H, Shapiro MJ, Zartler ER (2003) The effect of relaxation on the epitope mapping by saturation transfer difference NMR. J. Magn. Reson. 163:270–276.

6. McCullough C, Wang M, Rong L, Caffrey M (2012) Characterization of influenza hemagglutinin interactions with receptor by NMR. PLoS ONE 7:e33958.

7. Keller S, Vargas C, Zhao H, Piszczek G, Brautigam CA, Schuck P (2012) High-precision isothermal titration calorimetry with automated peak-shape analysis. Anal. Chem. 84:5066–5073.

8. Zhao H, Piszczek G, Schuck P (2015) SEDPHAT--a platform for global ITC analysis and global multi-method analysis of molecular interactions. Methods 76:137–148.

9. Paoletti F, Merzel F, Cassetta A, Ogris I, Covaceuszach S, Grdadolnik J, Lamba D, Golič Grdadolnik S (2021) Endogenous modulators of neurotrophin signaling: Landscape of the transient ATP-NGF interactions. Comput Struct Biotechnol J 19:2938–2949.

10. Ericsson UB, Hallberg BM, Detitta GT, Dekker N, Nordlund P (2006) Thermofluor-based high-throughput stability optimization of proteins for structural studies. Anal. Biochem. 357:289–298.

11. Blanchet CE, Spilotros A, Schwemmer F, Graewert MA, Kikhney A, Jeffries CM, Franke D, Mark D, Zengerle R, Cipriani F, et al. (2015) Versatile sample environments and automation for biological solution X-ray scattering experiments at the P12 beamline (PETRA III, DESY). Journal of Applied Crystallography 48:431–443.

12. Konarev PV, Volkov VV, Sokolova AV, Koch MHJ, Svergun DI (2003) *PRIMUS* : a Windows PC-based system for small-angle scattering data analysis. Journal of Applied Crystallography 36:1277–1282.

13. Franke D, Petoukhov MV, Konarev PV, Panjkovich A, Tuukkanen A, Mertens HDT, Kikhney AG, Hajizadeh NR, Franklin JM, Jeffries CM, et al. (2017) ATSAS 2.8: a comprehensive data analysis suite for small-angle scattering from macromolecular solutions. J Appl Crystallogr 50:1212–1225.

14. Guinier A (1939) La diffraction des rayons X aux très petits angles : application à l’étude de phénomènes ultramicroscopiques. Ann. Phys. 11:161–237.

15. Svergun DI (1992) Determination of the regularization parameter in indirect-transform methods using perceptual criteria. Journal of Applied Crystallography 25:495–503.

16. Porod G In: Small Angle X-ray Scattering. Academic Press; 1982. pp. 17–51.

17. Petoukhov MV, Svergun DI (2015) Ambiguity assessment of small-angle scattering curves from monodisperse systems. Acta Crystallographica Section D 71:1051–1058.

18. Petoukhov MV, Franke D, Shkumatov AV, Tria G, Kikhney AG, Gajda M, Gorba C, Mertens HDT, Konarev PV, Svergun DI (2012) New developments in the ATSAS program package for small-angle scattering data analysis. J Appl Cryst, J Appl Crystallogr 45:342–350.

19. Covaceuszach S, Konarev PV, Cassetta A, Paoletti F, Svergun DI, Lamba D, Cattaneo A (2015) The conundrum of the high-affinity NGF binding site formation unveiled? Biophys. J. 108:687–697.

20. Bernadó P, Mylonas E, Petoukhov MV, Blackledge M, Svergun DI (2007) Structural Characterization of Flexible Proteins Using Small-Angle X-ray Scattering. Journal of the American Chemical Society 129:5656–5664.

21. Lau AM, Claesen J, Hansen K, Politis A (2021) Deuteros 2.0: peptide-level significance testing of data from hydrogen deuterium exchange mass spectrometry. Bioinformatics 37:270–272.

22. Paoletti F, Covaceuszach S, Konarev PV, Gonfloni S, Malerba F, Schwarz E, Svergun DI, Cattaneo A, Lamba D (2009) Intrinsic structural disorder of mouse proNGF. Proteins 75:990–1009.

23. Nykjaer A, Lee R, Teng KK, Jansen P, Madsen P, Nielsen MS, Jacobsen C, Kliemannel M, Schwarz E, Willnow TE, et al. (2004) Sortilin is essential for proNGF-induced neuronal cell death. Nature 427:843–848.

24. Petoukhov MV, Franke D, Shkumatov AV, Tria G, Kikhney AG, Gajda M, Gorba C, Mertens HDT, Konarev PV, Svergun DI (2012) New developments in the ATSAS program package for small-angle scattering data analysis. J Appl Crystallogr 45:342–350.

25. Mizianty MJ, Peng Z, Kurgan L (2013) MFDp2. Intrinsically Disordered Proteins 1:e24428.

26. Ibáñez CF (1995) Neurotrophic factors: from structure-function studies to designing effective therapeutics. Trends Biotechnol 13:217–227.

**Figures and Tables**


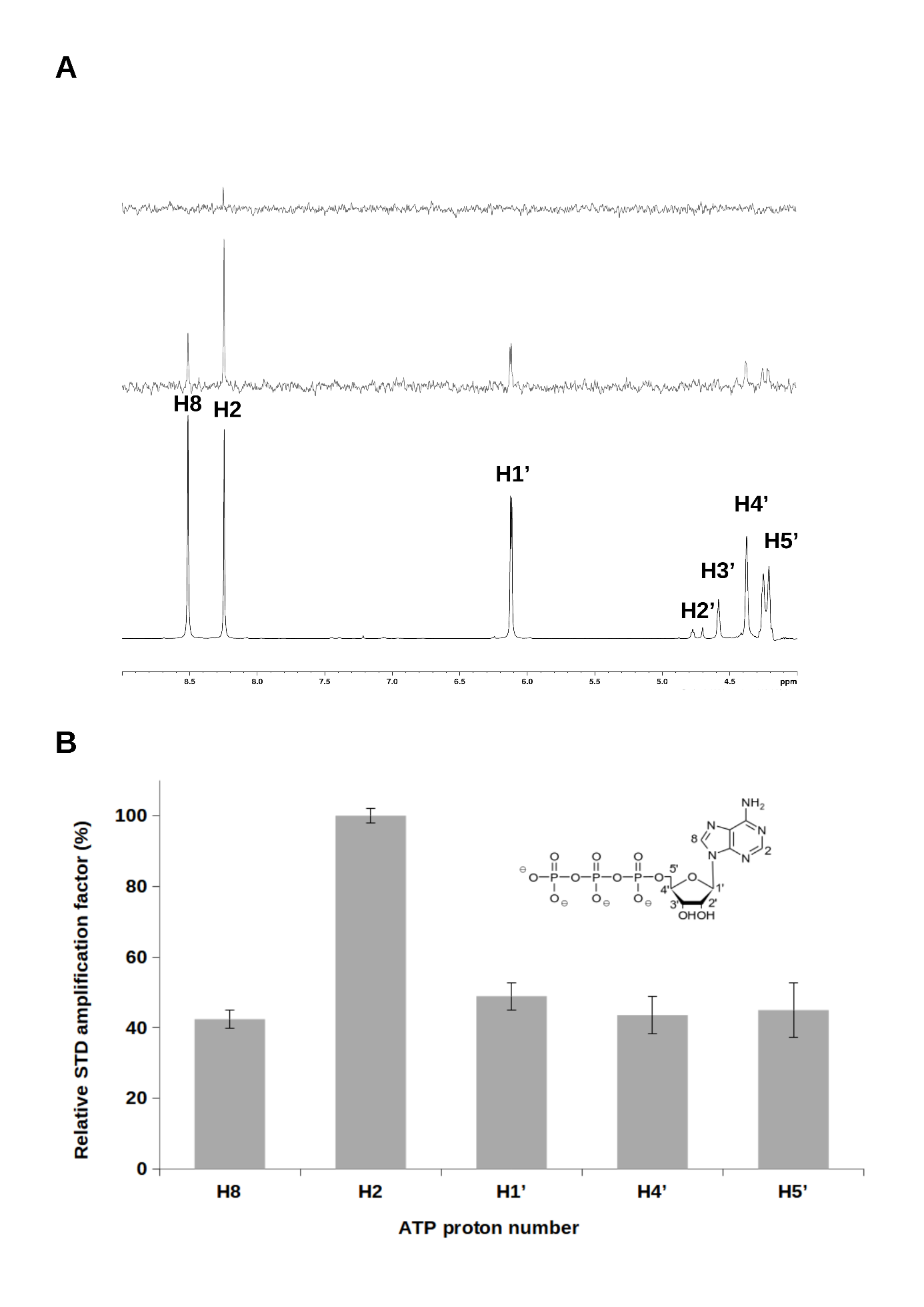


**Figure S1. Interaction of rh-proNGF with ATP monitored by 1D ^1^H Saturation Transfer Difference NMR experiment (^1^H STD-NMR)** (**A**) Reference spectrum (off resonance) (bottom) and STD spectrum (middle). The spectral intensities are not normalized. rh-proNGF concentration was 10 μM (with respect to the dimer), and the ratio ATP:rh-proNGF = 100:1. The sensitivity of the STD signals was strongly reduced under short-term saturation conditions, which were required to avoid the effect of the longitudinal relaxation rates on the signal intensities. In the STD control experiment on a sample with ATP without protein (top) only the residual signal for the H2 proton with the narrowest linewidth is observed. (**B**) Relative degree of saturation of individual protons of ATP determined from the 1D ^1^H STD NMR spectrum at 100-fold excess of ATP over rh-proNGF. The values are normalized to the intensity of the signal with the largest STD effect. The absence of certain STD amplification factors is due to low intensity of signals in STD spectra and does not represent a loss of interaction. The inset shows the ATP molecule with the proton-numbering scheme.


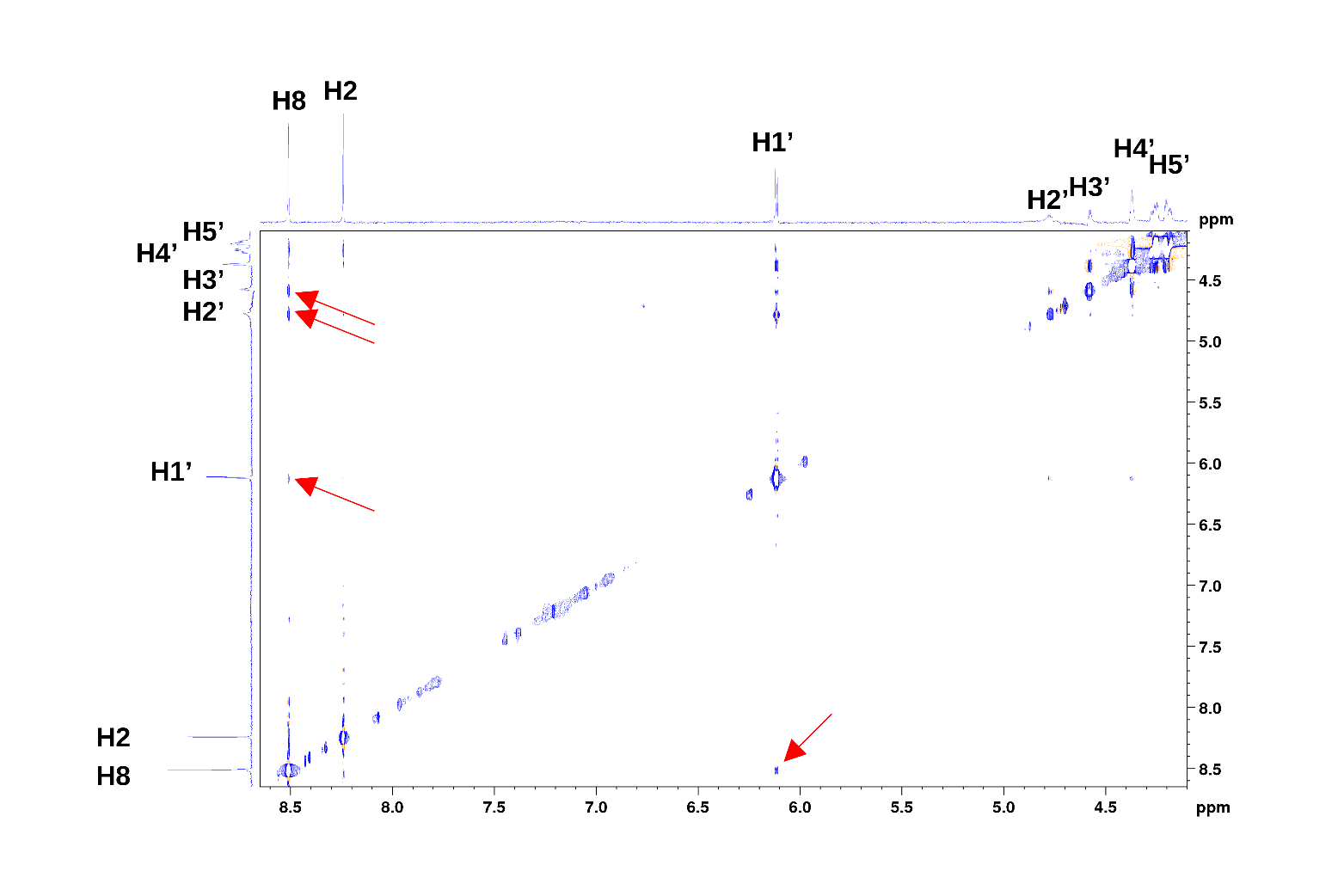


**Figure S2.** **Selected expansion from transferred NOESY (trNOESY) spectrum**. The non-trivial NOEs of ATP protons are marked by red arrows. The assignment of the ATP protons is also shown. The NOEs have the same sign as the diagonal peaks indicating the genuine binding of ATP to rh-proNGF.

**Figure S3.** **FT-IR of rh-proNGF in absence and in presence of ATP** - The infrared ATR spectra of rh-proNGF (black line) and rh-proNGF with ATP (blue line) after subtraction of the buffer spectrum. The blue spectrum is shifted up for clarity. The upshifted red line represents the difference between the black and blue spectra.


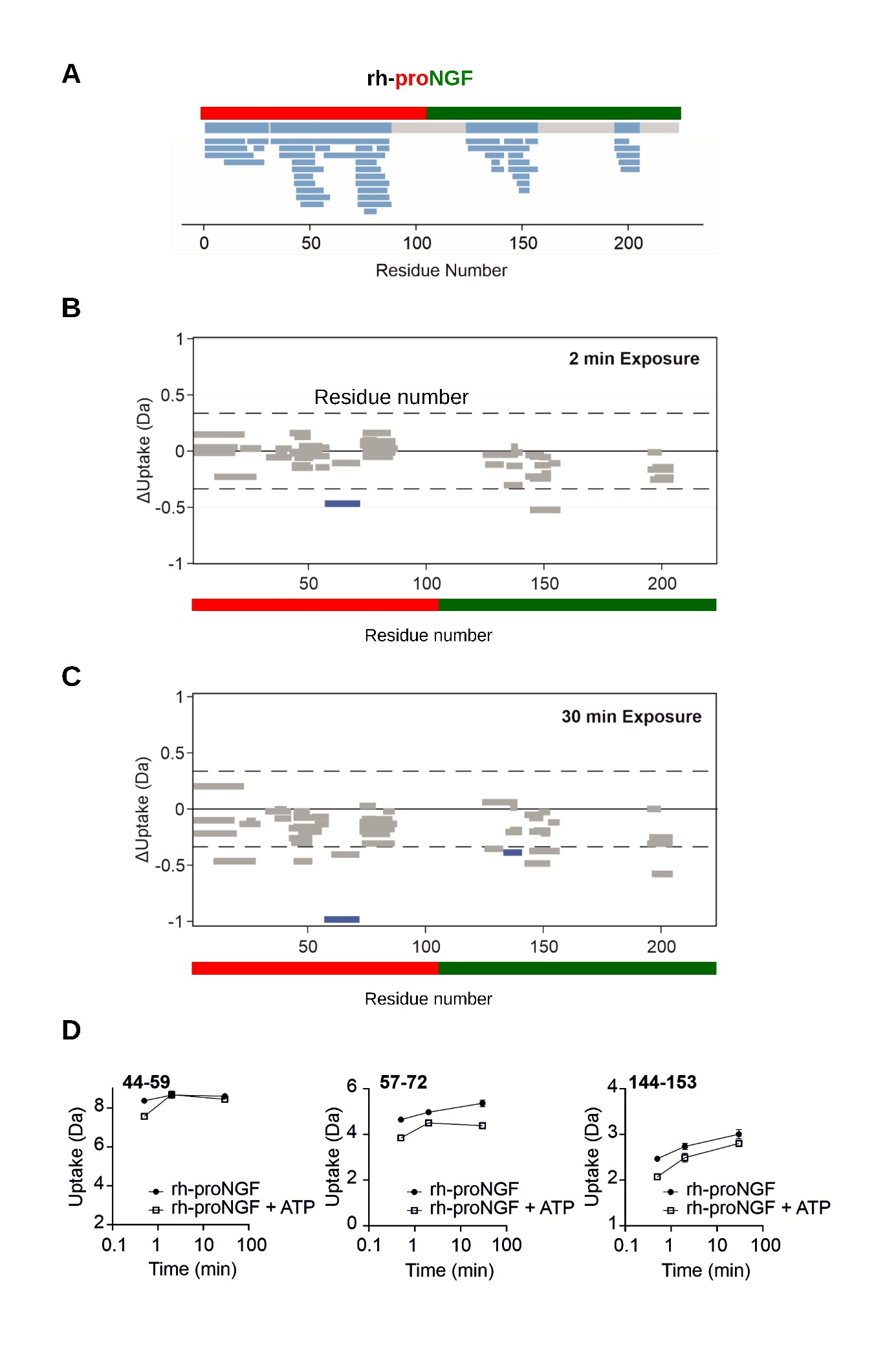


**Figure S4.** **HDX-MS data of rh-proNGF in the absence and presence of ATP.** (**A**) Peptide map showing detected peptides in rh-proNGF. The pro-peptide and mature NGF regions are represented as red and green bars, respectively. (**B, C**) Wood’s plots showing the differences in deuterium uptake in rh-proNGF, comparing rh-proNGF alone with rh-proNGF in the presence of ATP at 2 min and 30 min exposure to deuterium. Wood’s plots were generated using Deuteros ^21^. Peptides colored in blue are protected from exchange in the presence of ATP. Peptides with no significant difference between conditions, determined using a 99% confidence interval and a hybrid significance test (dotted line), are shown in grey. See Methods for experimental details. The pro-peptide and mature NGF regions are represented as red and green bars, respectively. (**D**) Representative deuterium uptake plots showing the extent of deuterium incorporation in three selected peptides in the absence and presence of ATP. Data are shown as mean ± standard deviation of three replicates. See Methods for experimental details.


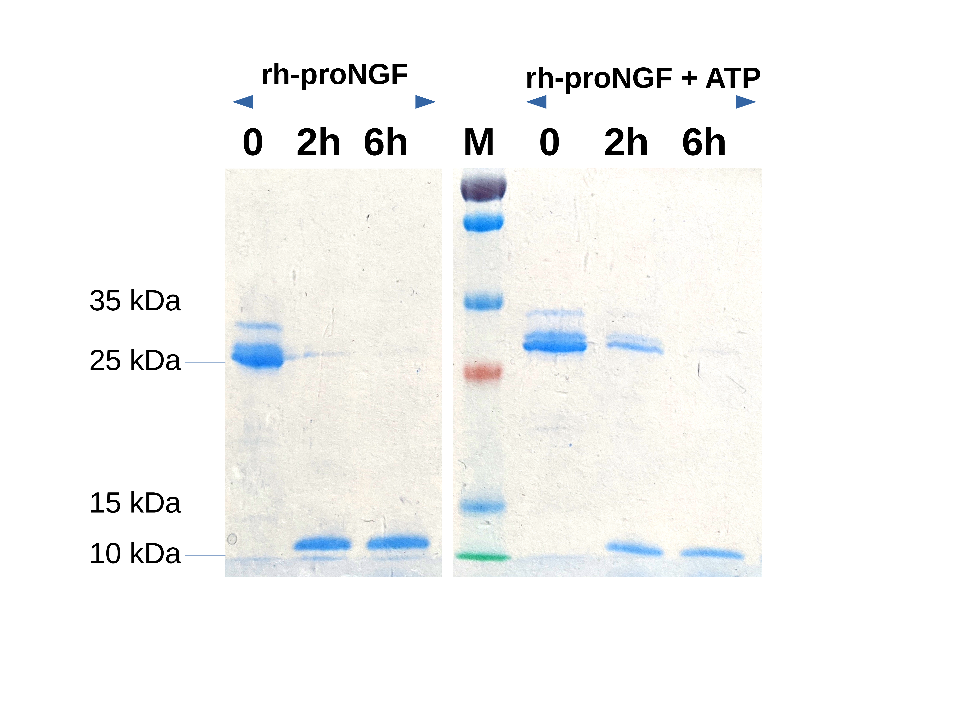


**Figure S5.** **rh-proNGF limited proteolysis with trypsin under controlled conditions**. 14% SDS-PAGE with rh-proNGF samples subjected to proteolysis with trypsin without or with ATP. Used concentrations: dimeric rh-proNGF: 8 µM; ATP: 240 µM.


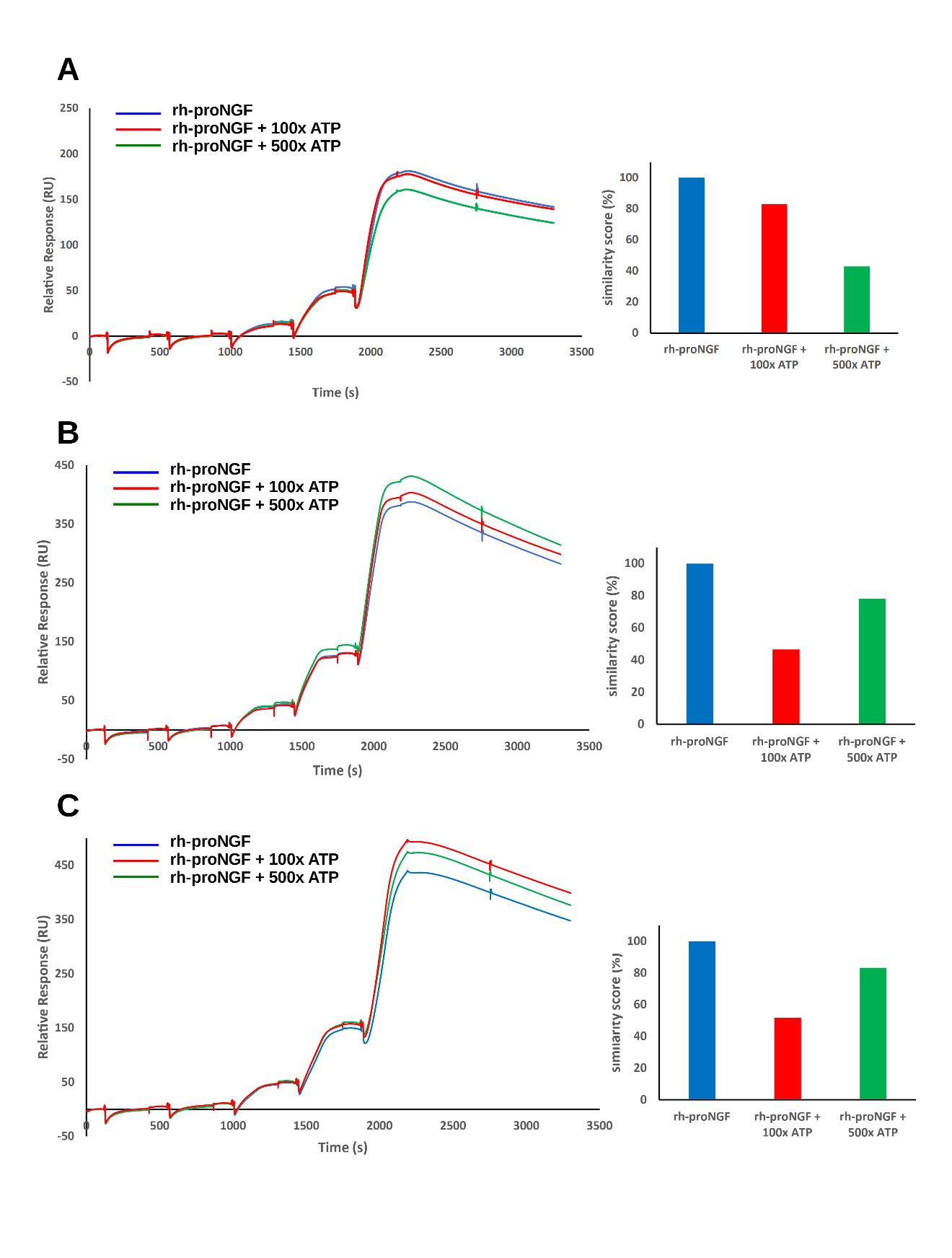


**Figure S6.** **Single Cycle Kinetics SPR sensorgrams in absence and in presence of different amounts of ATP.** The response over sortilin (**A**), p75^NTR^ (**B**) and TrkA (**C**) are reported. Blue trace: rh-proNGF, red trace: rh-proNGF with a 100-fold excess ATP; green trace: rh-proNGF with a 500-fold excess ATP. In each panel, on the left, the sensorgrams are reported, and on the right the plot of the similarity score (performed with the sensorgram comparison application of the BiaEvaluation software) of the curves, showing the level of similarity of the ATP-added samples sensorgrams to the reference rh-proNGF without ATP. (**A**) Sortilin shows a small difference in the binding intensity in absence or in presence of ATP, but a large excess of ATP induced a reduced binding intensity. (**B**) p75^NTR^ receptor response increases upon addition of ATP and shows a concentration-dependent response in the signal intensity. (**C**) In the case of TrkA, a large increase in the response intensity is observed in presence of ATP, but this effect is reversed when a large excess of ATP is used.


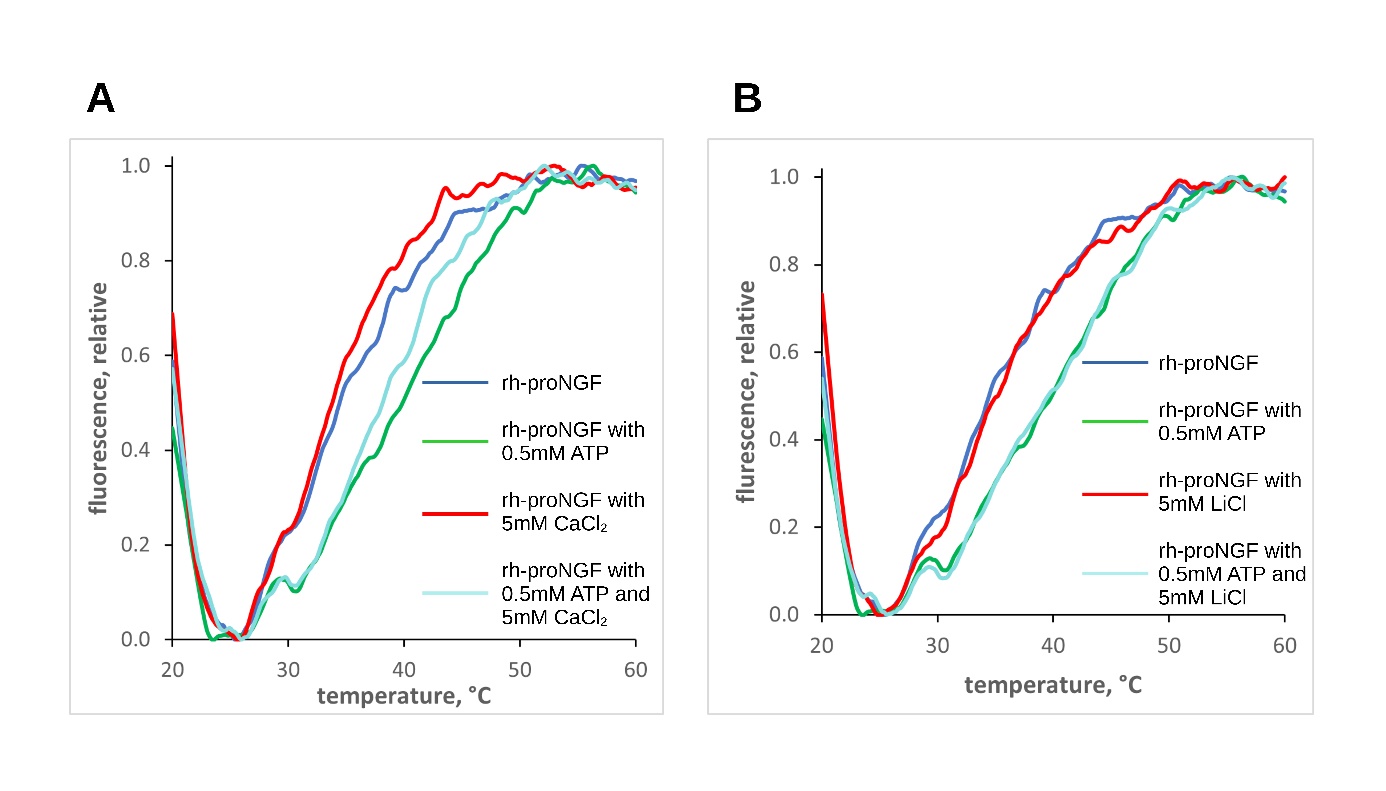


**Figure S7.** **DSF demonstrates that cations modulate ATP interaction with rh-proNGF.** **A**) Melting profiles of rh-proNGF in presence of 0.5 mM ATP, in the absence and in the presence of 5 mM Ca^2+^, respectively. **B**) Melting profiles of rh-proNGF in presence of 0.5 mM ATP, in the absence and in the presence of 5 mM Li^+^, respectively.

rh-proNGF concentration is 10 µM with respect to dimer. The curves are normalized according to the unfolding transition of the pro-peptide domain.


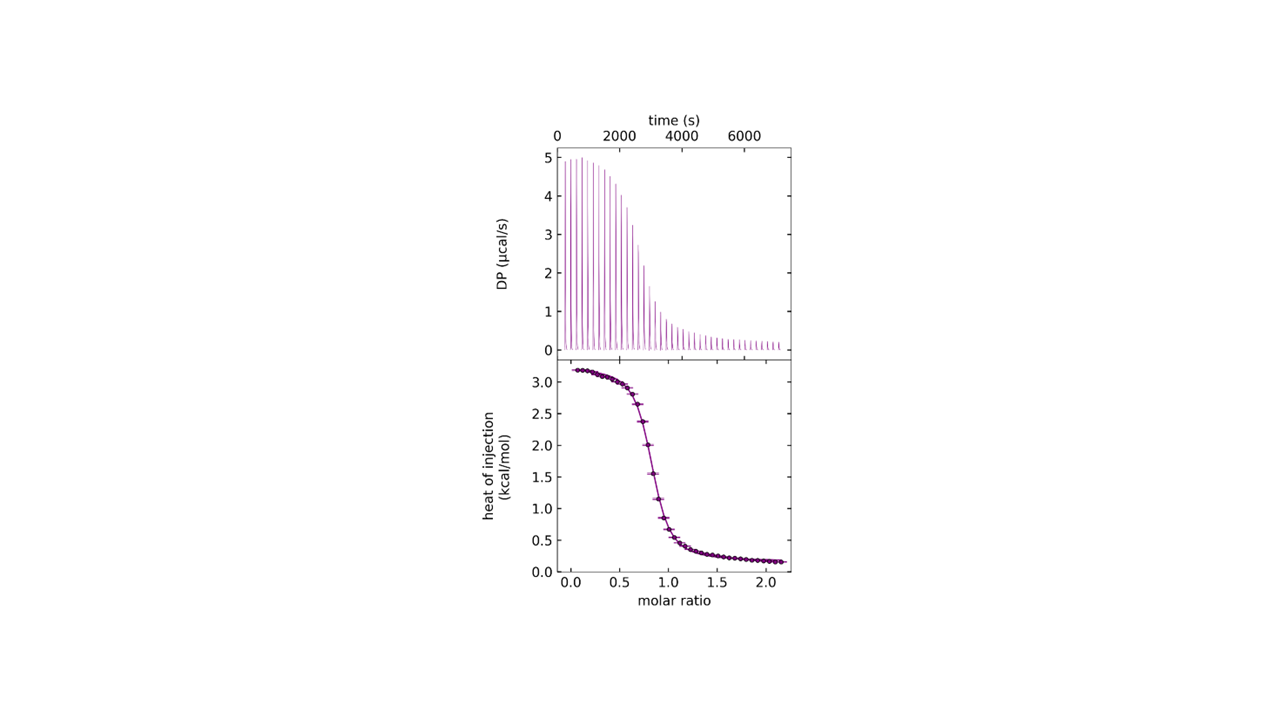


**Figure S8.** **Interaction of MgCl_2_ with ATP by ITC.** Thermograms and binding isotherms (lower panel) of a representative ATP/ MgCl_2_ titration (2 mM ATP, 20 mM MgCl_2_).


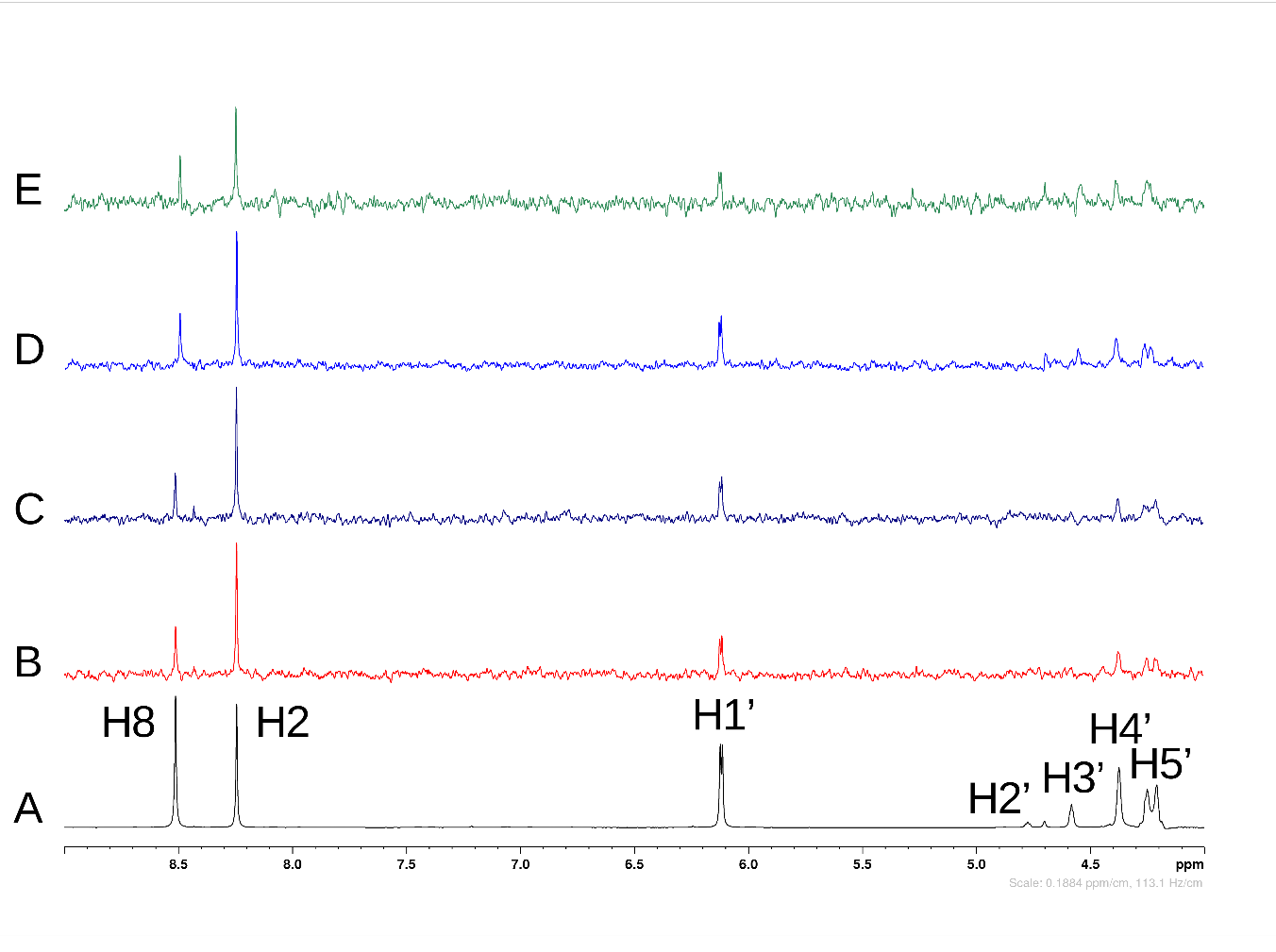


**Figure S9.** **Interaction of rh-proNGF with ATP by 1D ^1^H STD-NMR experiment**. Reference spectrum (off resonance) (**A**) and STD spectra at different Mg^2+^ concentrations (**B-E**). In all the STD spectra, ATP was added at 100-fold excess with respect to rh-proNGF dimer, that is a protein concentration of 10 µM (with respect to the dimer) and 1 mM ATP. The Mg^2+^ concentration in the STD spectra was the following: 0 µM (**B**), 10 µM (**C**), 1 mM (**D**), 2 mM (**E**). The spectra intensities are not normalized.

**Table S1.** **SAXS structural parameters**: radius of gyration (Rg), maximum dimension (Dmax) obtained from the p(r) distribution using GNOM; Porod volume (V_p_) and MM (Da), molecular mass (MM) estimated from comparison with I(0) intensity of the standard BSA sample.

| Data collection parameters | rh-proNGF + 1mM ATP |
| --- | --- |
| Instrument | P12 (PETRA III) |
| Beam geometry (mm^2^) | 0.2 x 0.12 |
| Wavelength (Å) | 1.24 |
| s range (Å^-1^) | 0.003–0.445 |
| Concentration range (mg/ mL) | 0.23-2.36 |
| Temperature (K) | 283 |
| Structural parameters |  |
| I(0) (A.U.) [from p(r)] | 12310±120 |
| Rg (A) [from p(r)] | 26.9±0.05 |
| I(0) (A.U.) [from Guinier] | 12533±123 |
| Rg (A) [from Guinier] | 27.3±0.05 |
| Dmax (Å) | 85±2 |
| Porod volume estimate (Å^3^) | 80500±4000 |
| Molecular mass determination (Da) |  |
| Molecular mass MM [from I(0)] | 47000±5000 |
| Molecular mass MM [from Porod volume] | 47000±5000 |
| Calculated monomeric MM from sequence | 25100 |
| Software employed |  |
| Primary data reduction | PRIMUS |
| Data processing | GNOM |
| Rigid body modeling | CORAL/EOM |
| Ambiguity estimate of 3D reconstruction | AMBIMETER |
| Three-dimensional graphic representations | PYMOL |

**Table S2.** Best fit valued obtained by global fitting of two independent ITC titrations of 20 mM MgCl_2_ titrations into 2 mM ATP.

| MgCl_2_ -> ATP | Best-fit value | 68.3% confidence interval |
| --- | --- | --- |
| K_D_ (μM) | 21.8 | 19.8 ; 24.1 |
| 𝛥H kcal Mol^-1^ | 3.07 | 3.04 ; 3.10 |
| 𝛥S cal Mol^-1^ K^-1^ | 31.4 |  |

**Table S3.** **HDX Data Summary Table.** SD = standard deviation, CI = confidence interval.

| Data Set | rh-proNGF | rh-proNGF + ATP |
| --- | --- | --- |
| HDX reaction details | 10 mM potassium phosphate, pD 7.0, 4 ºC | 10 mM potassium phosphate, pD 7.0, 4 ºC |
| HDX time course (min) | 0.5, 2, 30 | |
| HDX control samples | Maximally-labeled controls were not performed | |
| Back-exchange | ~ 30 % | |
| # of Peptides | 56 | 56 |
| Sequence coverage | 59.38% | 59.38% |
| Average peptide length / Redundancy | 10.73 / 4.52 | 10.73 / 4.52 |
| Replicates (biological or technical) | 3 (technical) | 3 (technical) |
| Repeatability (average SD) | 0.0626 | 0.0652 |
| Significant differences in HDX  (Δ HDX > X Da) | Reference state | Hybrid significance test: 99.0% CI: 0.34 Da / p-value < 0.01 |
